# Structures of phospho­nitrides in light of the extended Zintl–Klemm concept

**DOI:** 10.1107/S2052520625004263

**Published:** 2025-06-30

**Authors:** Angel Vegas, Hussien H. Osman, Alfonso Muñoz, Vladislav A. Blatov, Francisco Javier Manjón

**Affiliations:** a Universidad de Burgos Hospital del Rey s/n Burgos 09001 Spain; b Instituto de Diseño para la Fabricación y Producción Automatizada, MALTA Consolider Team Universitat Politècnica de València València 46022 Spain; c Instituto de Ciencia de los Materiales de laUniversitat de València MALTA Consolider Team Bujassot València 46100 Spain; d Chemistry Department, Faculty of Sciences Helwan University Cairo 11795 Egypt; e Departamento de Física, MALTA Consolider Team Universidad de La Laguna San Cristóbal de La Laguna Tenerife38200 Spain; f Samara State Technical University Molodogvardeyskaya St. 244 Samara 443100 Russian Federation; University of Geneva, Switzerland

**Keywords:** phospho­nitrides, crystal structures, crystal chemistry, extended Zintl–Klemm concept

## Abstract

The extended Zintl–Klemm concept (EZKC) is applied to explain the crystalline structures of phospho­nitrides (also known as nitridophosphates in the chemical literature). The examples of (AE)_2_AlP_8_N_15_(NH) (AE = Ca, Sr, Ba), Ge^IV^PN_3_ and *M*P_2_N_4_ (*M* = Be, Ca, Sr, Ba, Ge^II^) are mainly discussed, although the examples of LiGaGe and LiGaGeO_4_ have been also commented on due to their relation with BeP_2_N_4_. It is shown that the EZKC provides a better understanding of the structures of these compounds than in previous descriptions. In most of these nitrides, P atoms behave as pseudo-Si atoms and N atoms behave as pseudo-O atoms, so providing a good explanation for the four-connectivity of P atoms forming PN_4_ units, which behave as pseudo-SiO_4_ units like the SiO_4_ units in many polymorphs of SiO_2_. In addition, the EZKC shows that the notation of these compounds as phospho­nitrides is more appropriate than as nitridophosphates because N atoms act as the anions in these compounds.

## Abstract 2

The extended Zintl–Klemm concept (EZKC), applied satisfactorily to rationalize the structures of aluminates and silicates, also accounts for the structures of ternary phospho­nitrides.

## 1. Introduction

In recent decades, there has been a notable increase in interest in metal phospho­nitrides, largely due to their significant physical properties and potential applications. A significant proportion of these materials are ultra hard, and in addition, exhibit luminescent properties when doped with Eu^2+^. For illustrative purposes, it is worth including the series of ternary compounds *M*P_2_N_4_ (*M* = Be, Ca, Sr, Ba, Mn, Cd) (Karau & Schnick, 2005 ▸; Karau *et al.*, 2007 ▸; Pucher *et al.*, 2015 ▸) and the recently reported Ge^II^P_2_N_4_ (de Boer *et al.*, 2023 ▸) and Ge^IV^PN_3_ (Ambach *et al.*, 2024 ▸) compounds. In addition to the mentioned compounds, the recently reported and promising quaternary phospho­nitrides (AE)_2_AlP_8_N_15_(NH) (AE = Ca, Sr, Ba) can be included in the list (Pointner *et al.*, 2024 ▸).

Many articles concerning metal phospho­nitrides emphasize the synthesis methods, which are predominantly conducted under high-pressure (HP) conditions. Additionally, the physical properties of these compounds and the theoretical calculations designed to elucidate their luminescent properties are frequently discussed. However, the crystal structures, described with the classical cation-centered anionic polyhedra, have provided only limited insight into explaining the varying coordination numbers (CNs) of each atom species. For instance, the structures of (AE)_2_AlP_8_N_15_(NH) (AE = Ca, Sr, Ba) compounds have been described as comprising three structural motifs (Pointner *et al.*, 2024 ▸). First, there are three-dimensional tetrahedral PN_4_ networks. Second, there are irregular AE-centered N polyhedra. Third, there are non-condensed (isolated) AlN_6_ octahedra. In this context, an elucidation of the factors responsible for this diversity of structural elements would have been desirable; however, it has not been addressed to our knowledge. In particular, it has not been explained why the Al atoms are hexacoordinated (isolated AlN_6_ octahedra) in (AE)_2_AlP_8_N_15_(NH) (Pointner *et al.*, 2024 ▸), whereas the P atoms are tetrahedrally coordinated in *M*P_2_N_4_ (*M* = Be, Ca, Sr, Ba) (Karau & Schnick, 2005 ▸; Karau *et al.*, 2007 ▸; Pucher *et al.*, 2015 ▸) as well as in Ge^II^P_2_N_4_ (de Boer *et al.*, 2023 ▸) and Ge^IV^PN_3_ (Ambach *et al.*, 2024 ▸). The cation/anion ratios (P/N ratio for example) cannot account for such different behaviors.

Challenging features also appear in the series of metal phospho­nitrides *M*P_2_N_4_ (*M* = Be, Ca, Sr, Ba, Mn, Cd) (de Boer *et al.*, 2023 ▸). For example, BeP_2_N_4_ is phenakite-like at ambient conditions of temperature and pressure (Pucher *et al.*, 2010 ▸); a structure containing a three-dimensional four-connected network of PN_4_ tetrahedra, which transforms into an ultra-incompressible material of the spinel type at high-temperature and high-pressure conditions with P atoms in octahedral coordination (Vogel *et al.*, 2020 ▸). Curiously, the remaining compounds in the series have more open, four-connected P skeletons, analogous to those found in oxo-aluminates and aluminosilicates (Santamaría-Pérez & Vegas, 2003 ▸).

In the same way, there are remarkable and still unexplained differences between the P skeletons of the related compounds Ge^II^P_2_N_4_ (de Boer *et al.*, 2023 ▸) and Ge^IV^PN_3_ (Ambach *et al.*, 2024 ▸). In Ge^II^P_2_N_4_, the P skeleton is revealed as a four-connected network, analogous to the Si skeletons of the phases of SiO_2_ at room pressure. Conversely, in Ge^IV^PN_3_, the P skeleton forms zigzag chains of corner-connected PN_4_ tetrahedra in which the P substructure is formed by planar chains of two-connected P atoms.

We consider that these seemingly strange connectivities between the P atoms are not capricious and must obey a law that accounts for such structural diversity. An example of how such unexpected behavior can be rationalized can be found in the works of Vegas and coworkers (Santamaría-Pérez & Vegas, 2003 ▸; Santamaría-Pérez *et al.*, 2005 ▸), in which all aluminates and silicates with Al and Si skeletons were put on a common basis by applying the extended Zintl–Klemm concept (hereafter EZKC) to the cation subarrays of these two families of compounds. This concept was extended to many other compounds in subsequent works (Vegas *et al.*, 2009 ▸; Vegas, 2018 ▸).

Here, we analyze the structures of several phospho­nitrides in the context of the EZKC to provide a rational explanation for their tetrahedral P skeletons, as well as for the unexpected octahedral coordination adopted by the P atoms in some of them. The main purpose of this work is to show that the EZKC concept, successfully applied to rationalize the structures of aluminates (Santamaría-Pérez & Vegas, 2003 ▸), silicates (Santamaría-Pérez *et al.*, 2005 ▸), and the germanate (NH_4_)_2_Ge^[6]^[Ge^[4]^_6_O_15_] (Vegas & Jenkins, 2017 ▸), allows us to account for the topology and connectivity of the P skeletons in phospho­nitrides, a feature that cannot be explained with the traditional ionic model. We will show that these compounds obey a unique principle that explains all their diverse atomic connectivity.

In Table 1 ▸ we have summarized the compounds that are discussed in this work, along with their formulae, the topological name of their structures, the pseudo-formulae obtained by applying the EZKC, and the corresponding references.

## 2. Description of the crystal structures

### 2.1. (AE)_2_AlP_8_N_15_(NH) (AE = Ca, Sr, Ba) (orthorhombic, *Pnma*, No. 62)

This series of phospho­nitrides (also known as imido­nitridophosphates) was reported by Pointner *et al.* (2024 ▸). In that paper, the authors emphasize three matters relative to their structures: (i) concerns the existence of a tetrahedra network, formed by condensed PN_4_ tetrahedra; (ii) refers to the presence of (AE)-centered coordination polyhedra; (ii) notes the existence of isolated, non-condensed AlN_6_ octahedra. We have not found, however, any discussion regarding the correlation between the three structural moieties.

**Figure 1:**
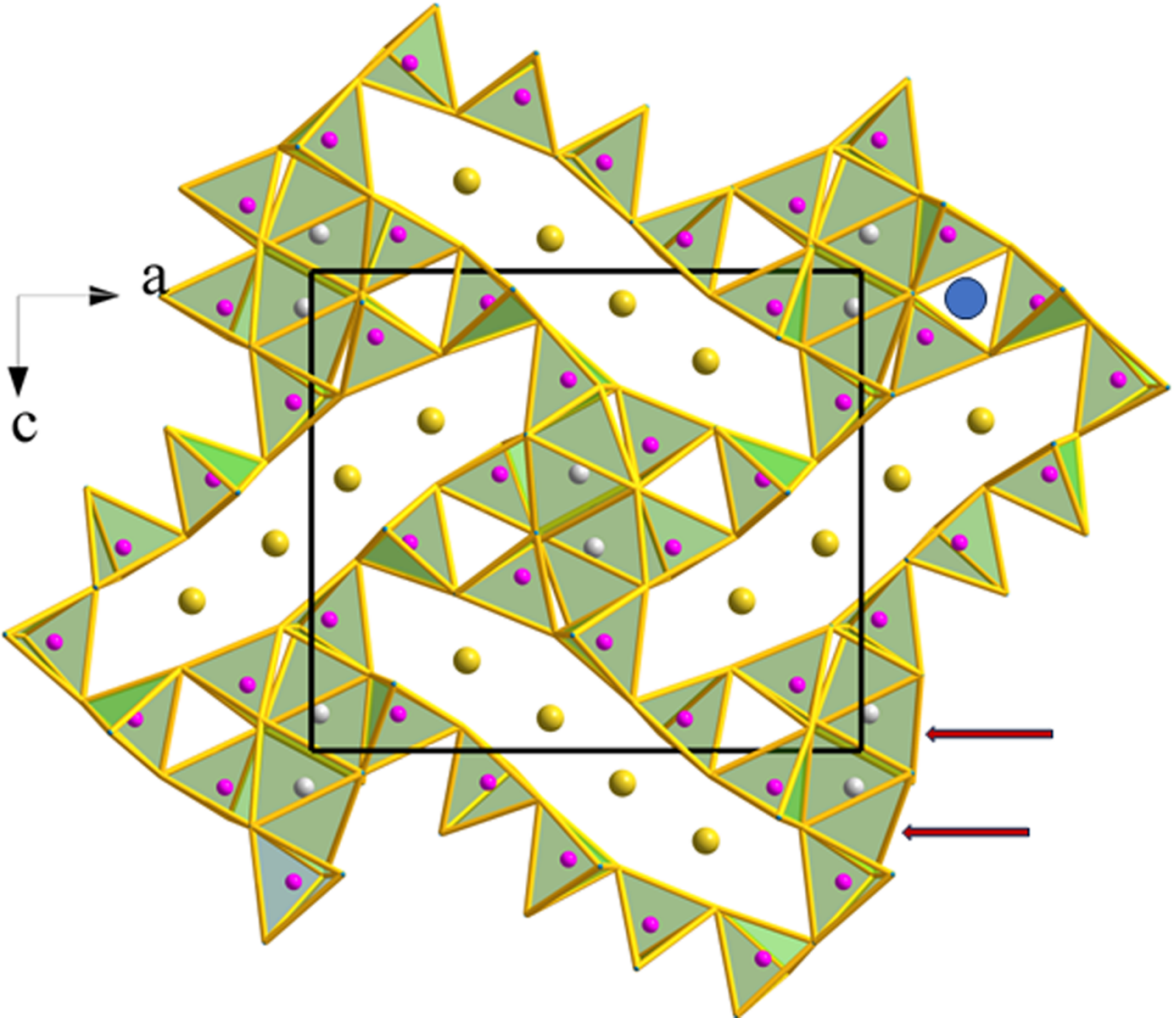
Orthorhombic structure of Ca_2_AlP_8_N_15_(NH) viewed in the *ac* plane. Ca: yellow, P: violet, N: blue, Al: light gray. The pairs of AlN_6_ octahedra, marked with arrows, close in projection, do not share any edges, but all PN_4_ tetrahedra are connected by sharing vertices, forming a three-dimensional network. A blue circle is drawn at the center of three tetrahedra sharing vertices (upper-right side).

Fig. 1 ▸ shows the orthorhombic *Pnma* (No. 62) structure of these phospho­nitrides projected on the *ac* plane. It is formed by a very complicated arrangement of PN_4_ tetrahedra leaving very elongated tunnels, in which groups of four (AE) atoms are located (see yellow spheres in Fig. 1 ▸). The P atoms forming the elongated spaces, in turn form an elongated P_12_ ring. The pairs of AlN_6_ octahedra are also visible in Fig. 1 ▸ at the corners and at the central part of the unit cell. Two of them are indicated with arrows. Even if they seem to be close to each other in projection, there is no connection between them. Further, these pairs of AlN_6_ octahedra are surrounded by six-membered rings of PN_4_ tetrahedra. Finally, three-membered (PN_4_)_3_ rings are also visible. One of them is marked by a blue circle in Fig. 1 ▸.

After this description in terms of classical cation-centered anionic polyhedra, we will tackle the description of Ca_2_AlP_8_N_15_(NH) in terms of the EZKC, followed by topological analysis of its P skeleton.

The EZKC involves the electron transfer between atoms, typically from the more electropositive to the more electronegative atoms. It can be considered that, in Ca_2_AlP_8_N_15_(NH), the N atoms receive four electrons from the two Ca atoms, three from the Al atom, and eight electrons from the eight P atoms. Thus, there are 15 electrons donated to 15 N atoms, so that N atoms behave as pseudo-O (Ψ-O) atoms, P atoms behave as pseudo-Si (Ψ-Si) atoms, Al atoms behave as pseudo-Ne (Ψ-Ne) atoms, and Ca atoms behave as pseudo-Ar (Ψ-Ar) atoms. As in other networks consistent with the EZKC, and according to the 8-N rule, the P(Ψ-Si) skeleton is four-connected in Ca_2_AlP_8_N_15_(NH), whereas Al^3+^ cations (Ψ-Ne) adopt an octahedral coordination, according to their donor character (see Santamaría-Pérez *et al.*, 2005 ▸), and Ca^2+^ cations (Ψ-Ar) adopt a variable CN, as already pointed out by Pointner *et al.* (2024 ▸). According to the EZKC, the compound can then be reformulated as (Ψ-Ar)_2_(Ψ-Ne)[Ψ-SiO_2_]_8_.

**Figure 2:**
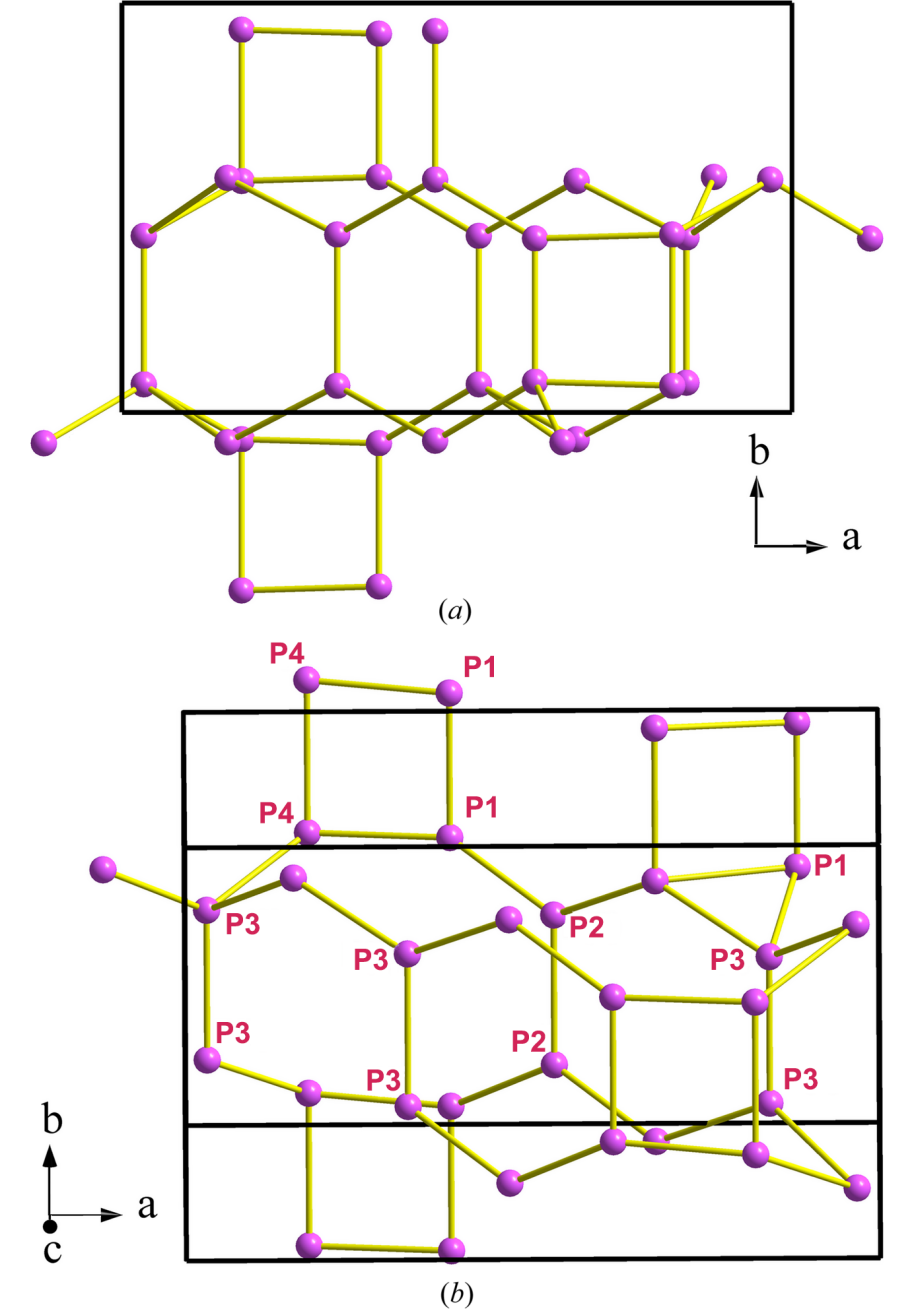
(*a*) Part of the P(Ψ-Si) skeleton in Ca_2_AlP_8_N_15_(NH) showing the four-connectivity of the Ψ-Si atoms. Note the sequence of 4-8-4 rings parallel to the *b* axis as well as the series of six-membered rings in boat conformation that are parallel to the *a* axis. (*b*) Another view of the same skeleton showing the 12-membered ring formed by the 3-4-1-2-4-3-3-4-1-3-3-2 P atoms. The elongated 12-membered ring is that embedding the four Ca atoms, also drawn in Fig. 1 ▸.

The P skeleton in Ca_2_AlP_8_N_15_(NH) forms a 4^4^-coordinated net with the point symbol {3.4.5.6^2^.7}_2_{3.6^5^}{6^6^}; we have deposited it into the *TopCryst* database (Shevchenko *et al.*, 2022 ▸) under the name 4^4^T170. Two views of this P skeleton are depicted in Fig. 2 ▸. They show the different rings existing in the P skeleton, *i.e.* three-, four-, six-, eight- and 12-membered rings. Some ring’s connections, like the extended sequence of 4-8-4 rings, running parallel to the *b* axis [see Fig. 2 ▸(*a*)], resemble the same motif existing in the structures of CrB_4_, AlPO_4_·2H_2_O (metavariscite), CaB_2_C_2_, CaAl_2_Si_2_O_8_ (anortite), HP-CuCl and Ba[Al_2_Si_2_O_8_] (paracelsian); the latter being a variant of the same skeleton (Vegas, 2018 ▸; chapters 8, 12–14; Shevchenko *et al.*, 2022 ▸).

**Figure 3:**
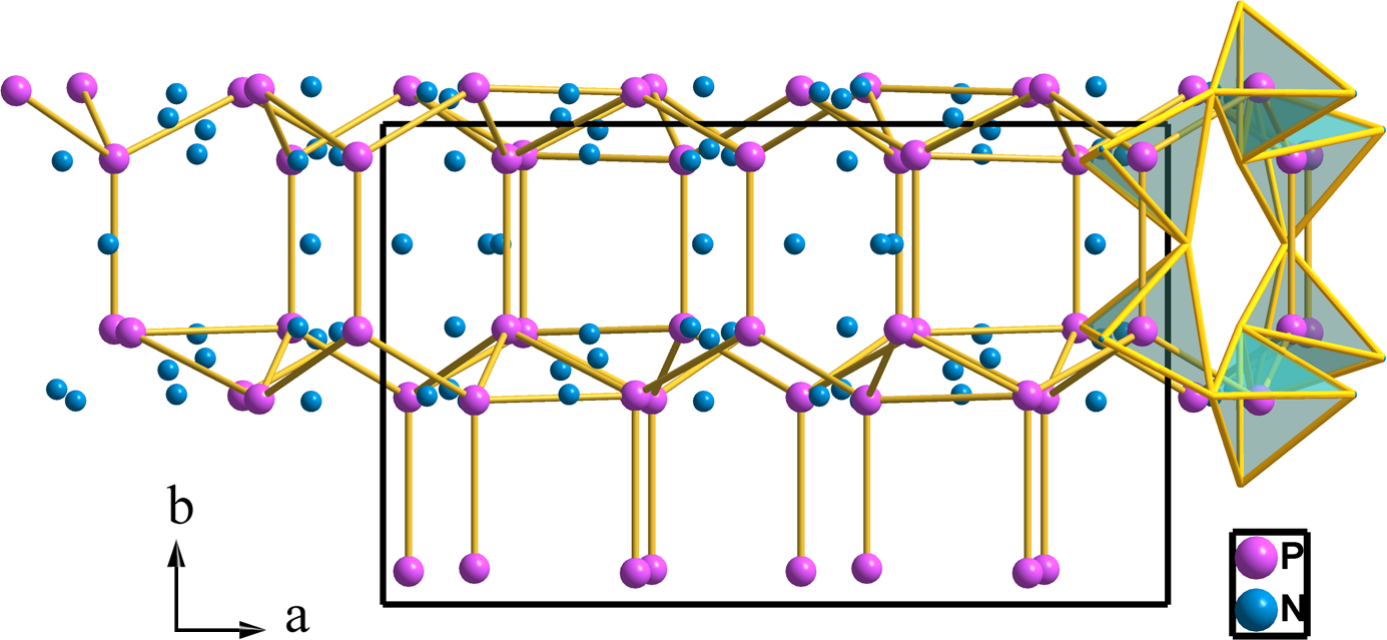
Two-connected layers of P (Ψ-Si) atoms in Ca_2_AlP_8_N_15_(NH) that are perpendicular to the *b* axis. They are projected on the *ab* plane. Each layer is formed by three-, six- and 12-membered rings in which P atoms are three-connected, but they become four-connected by forming one additional contact with P atoms in adjacent layers. The new rings between layers are of two types: hexagonal boat-conformation and squares. In one of the P_6_ rings with boat conformation (upper-right side), we have drawn the PN_4_ (Ψ-SiO_4_) tetrahedra. P: violet; N: blue.

The partial skeleton depicted in Fig. 3 ▸ can help us to understand better the amazing structure of Ca_2_AlP_8_N_15_(NH). It is composed of layers of puckered three-, six- and 12-membered rings. Within one sheet, each P atom is connected to three neighboring P atoms. The fourfold P-atom connectivity is achieved when each P atom also connects with one P atom from an adjacent layer. The interlayer contacts yield new six-membered boat-conformed vertical rings. Therefore, the N (Ψ-O) atoms are located near the middle point of each P–P contact, thus forming the three-dimensional network of PN_4_ (Ψ-SiO_4_) tetrahedra. In summary, we want to highlight that the structure of these phospho­nitrides with the tetrahedral coordination of P atoms can be explained in terms of the EZKC.

This point is important because to explain any structure one must be able to account for the connectivity of the atoms forming the main skeleton (in this case, the P skeleton). If the P atoms are four-connected, that means that the P atoms behave as if they were Si atoms and this character is only explained by assuming that the P atoms convert into Ψ-Si atoms by transferring one electron each to the N atoms which, in turn, become Ψ-O, as indeed is proposed by the EZKC.

### 2.2. Ge^IV^PN_3_ (monoclinic, *C*2/*c*, No. 15)

**Figure 4:**
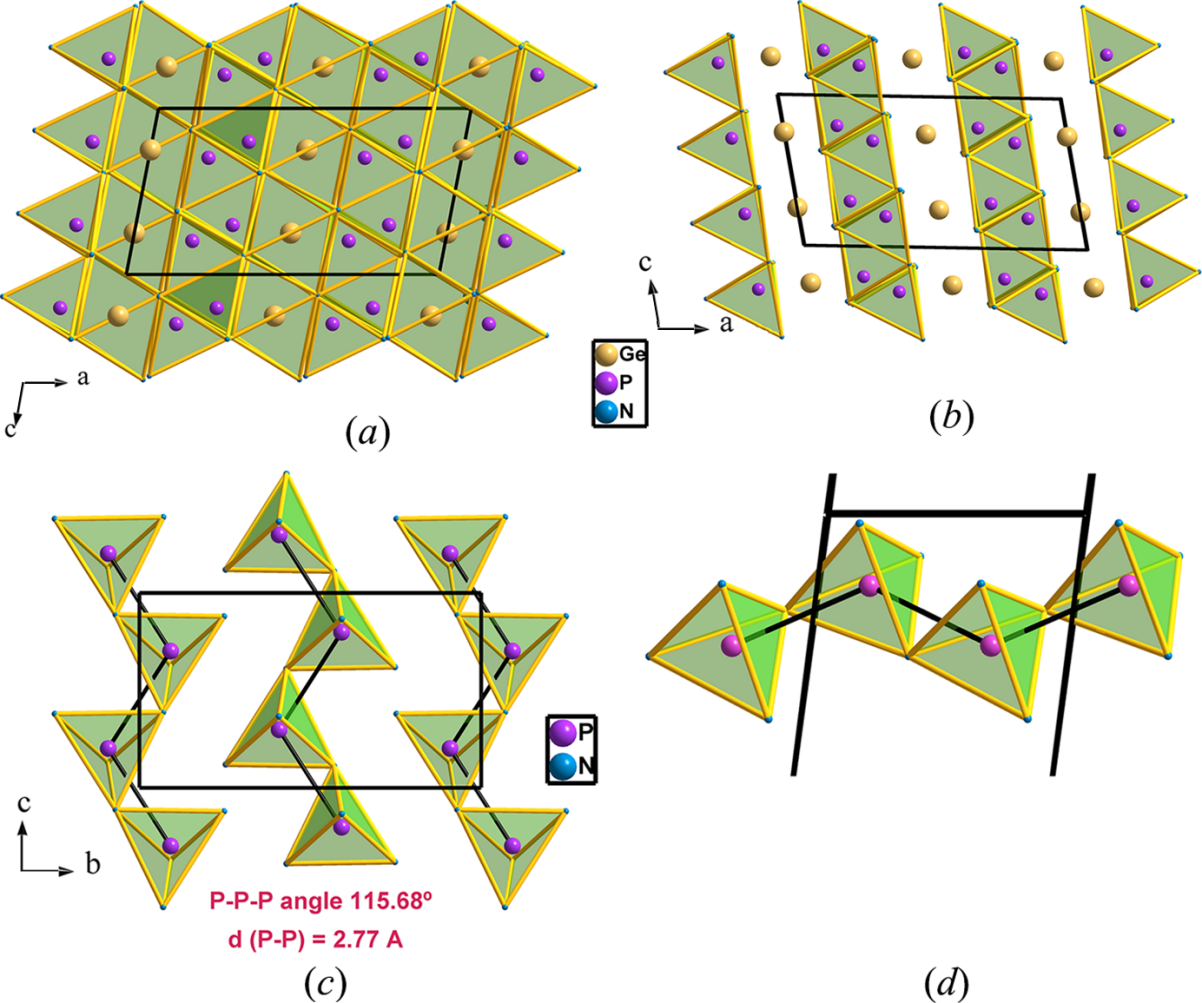
(*a*) Structure of Ge^IV^PN_3_, projected on the *ac* plane, representing the chains of GeN_6_ octahedra that alternate with the zigzag tetrahedral chains (*zweier*), which are drawn solely in (*b*), intercalated with the Ge atoms. (*c*) The same structure projected onto the *bc* plane. The drawing represents only the chains of PN_4_ tetrahedra (Ψ-SO_4_). The P atoms are connected by black lines to show that the P(Ψ-S) atoms form chains similar to those of the real S atoms. (*d*) One isolated chain of PN_4_ tetrahedra shows its similarity with the chains represented in Fig. 5 ▸.

Ge^IV^PN_3_ was synthesized at 44.4 GPa (Ambach *et al.*, 2024 ▸) and its structure shows a quite different scenario. The structure was described as formed by alternating layers of GeN_6_ octahedra and PN_4_ tetrahedra along [100] [see Figs. 4 ▸(*a*) and 4 ▸(*b*)]. Each layer of GeN_6_ octahedra consists of double zigzag chains of edge-sharing GeN_6_ octahedra with no interconnection between the chains. In turn, the tetrahedral layers are built up of chains of condensed PN_4_ tetrahedra (*zweier* single chains). Each tetrahedron shares two vertices with the two contiguous ones forming a [PN_3_]^4−^ polyanion. Within the tetrahedral chains, the P atoms form planar two-connected zigzag chains as depicted in Figs. 4 ▸(*c*) and 4 ▸(*d*).

Interestingly, the structure permits an alternative description in terms of the EZKC. If we consider that the Ge atom acts as a donor and transfers one electron to the P atom, the latter transforms into Ψ-S. If in addition, the remaining three valence electrons of the Ge atom are transferred to the three N atoms, they transform into Ψ-O atoms. All in all, this results in the stoichiometry (Ψ-Zn^2+^)[Ψ-SO_3_], whose crystal structure, projected on the *ac* plane in Fig. 4 ▸, will be analyzed next.

The *zweier* single chains of corner-sharing PN_4_ tetrahedra, alternating with chains of edge-sharing GeN_6_ octahedra are depicted in Fig. 4 ▸(*a*). Both motifs form blocks parallel to the (001) plane. The planes of Ge atoms become visible, alternating with the PN_4_ chains when the N_6_ octahedra are omitted [see Fig. 4 ▸(*b*)]. If the Ge atoms are neglected, the PN_4_ chains appear isolated, projected onto the *bc* plane, as depicted in Fig. 4 ▸(*c*). One of the PN_4_ chains is shown isolated in Fig. 4 ▸(*d*).

**Figure 5:**
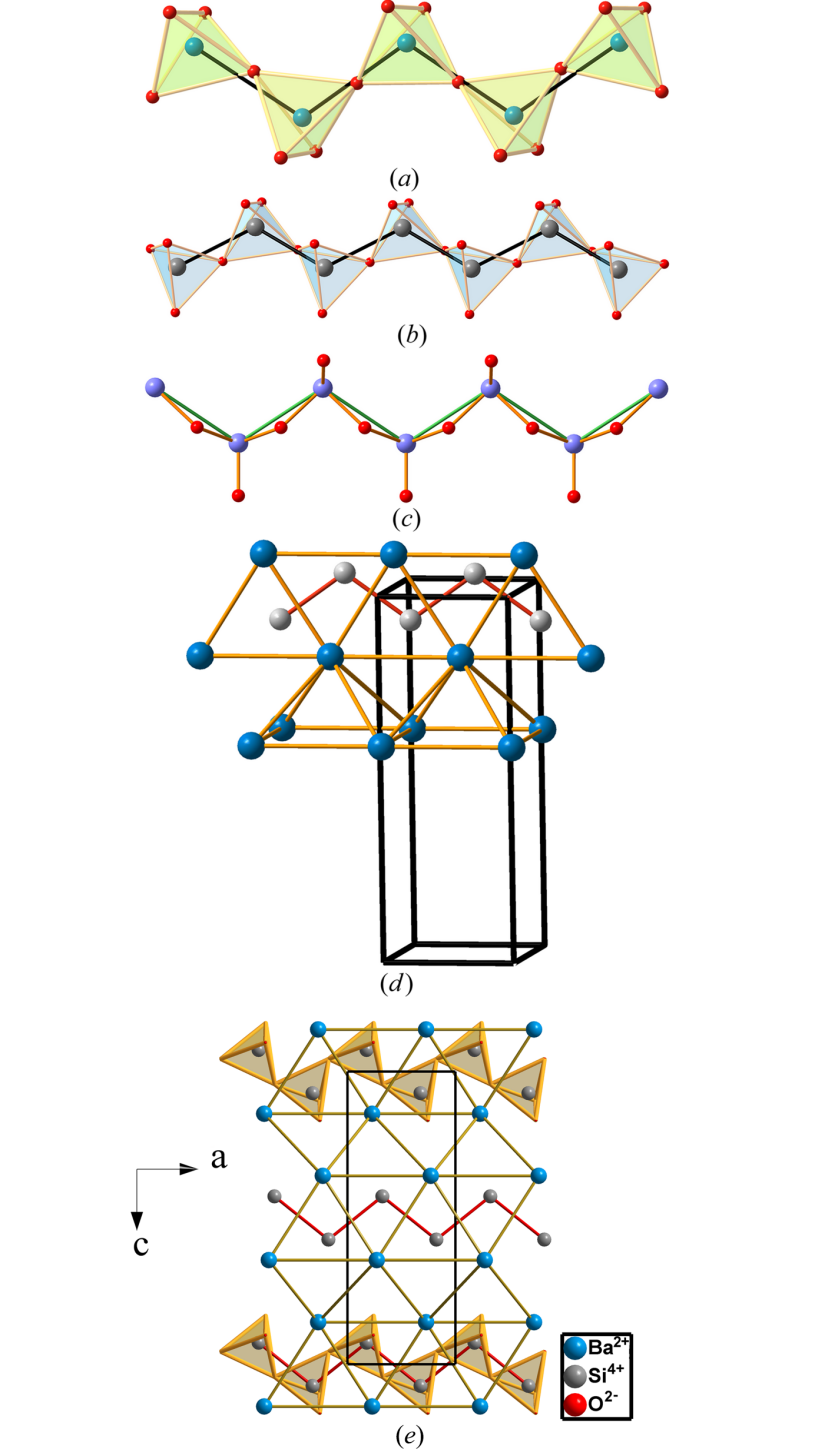
(*a*) Fragment of the planar chain of fibrous SO_3_ formed by SO_4_ corner-sharing tetrahedra. The S atoms show the twofold connectivity characteristic of sextels. S: green, O: red. (*b*) Structure of the polyanion [Si_2_O_6_]^4−^ (Ψ-SO_3_) in the silicate Na_4_[Si_2_O_6_]. Si: dark gray, O: red. (*c*) Fragment of the planar chain of SeO_2_(**E**) ≡ SeO_3_. Se: light-purple; O: red. (*d*) Perspective view of a fragment of the structure of the Zintl phase BaSi (B33 type; *Cmcm*, No. 63), which shows the same zigzag chains of the Si (Ψ-S) atoms. Ba: dark blue; Si: light gray. (*e*) The structure of BaSiO_3_ (*P*2_1_2_1_2_1_, No. 19), where the [BaSi] substructure adopts the same topology as the Zintl phase BaSi (*d*) despite being embedded in an O-atom matrix. Views (*a*), (*b*), (*c*) and (*d*) are reproduced from Vegas (2018 ▸) with permission.

Since the P atoms are converted into Ψ-S, they are two-connected as characteristic of sextels, forming extended planar chains of P atoms separated at distances of 2.77 Å, with P—P—P angles of 115.68°. This motif is quite frequent in the extended polyanions in the structures of aluminates, silicates and phosphates, some of which are represented in Fig. 5 ▸. They correspond to: (*a*) fragment of a planar zigzag chain in real SO_3_; (*b*) the structure of the polyanion [Si_2_O_6_]^4−^ ≡ (Ψ-SO_3_) as an unbranched single chain in the silicate Na_4_{***uB***,2,1^1^_∞_}[Si_2_O_6_] (Santamaría-Pérez *et al.*, 2005 ▸); (*c*) The structure of SeO_2_(**E**) ≡ SeO_3_ (Vegas, 2018 ▸), where the presence of the LEP **(E)** on the Se atoms means that SeO_2_(**E**) can be formulated as Ψ-SeO_3_; (*d*) the fragment of the B33 structure of the Zintl phase BaSi in which the Si atoms (Ψ-S) form similar chains with the Si substructure in [Si_2_O_6_]^4−^ (Ψ-SO_3_) and in SeO_2_(**E**) in Figs. 5 ▸(*b*) and 5 ▸(*c*), respectively; (*e*) the structure of BaSiO_3_ in which the [BaSi] partial structure is the same as that of BaSi (Rieger & Parthé, 1964 ▸) [Fig. 5 ▸(*d*)], even though in BaSiO_3_ the Si atoms are tetrahedrally coordinated by four O atoms, forming the extended SiO_3_ chains similar to those of Na_4_Si_2_O_6_, drawn in Fig. 5 ▸(*b*).

### 2.3. Ge^II^P_2_N_4_ (orthorhombic, *Pna*2_1_, No. 33)

**Figure 6:**
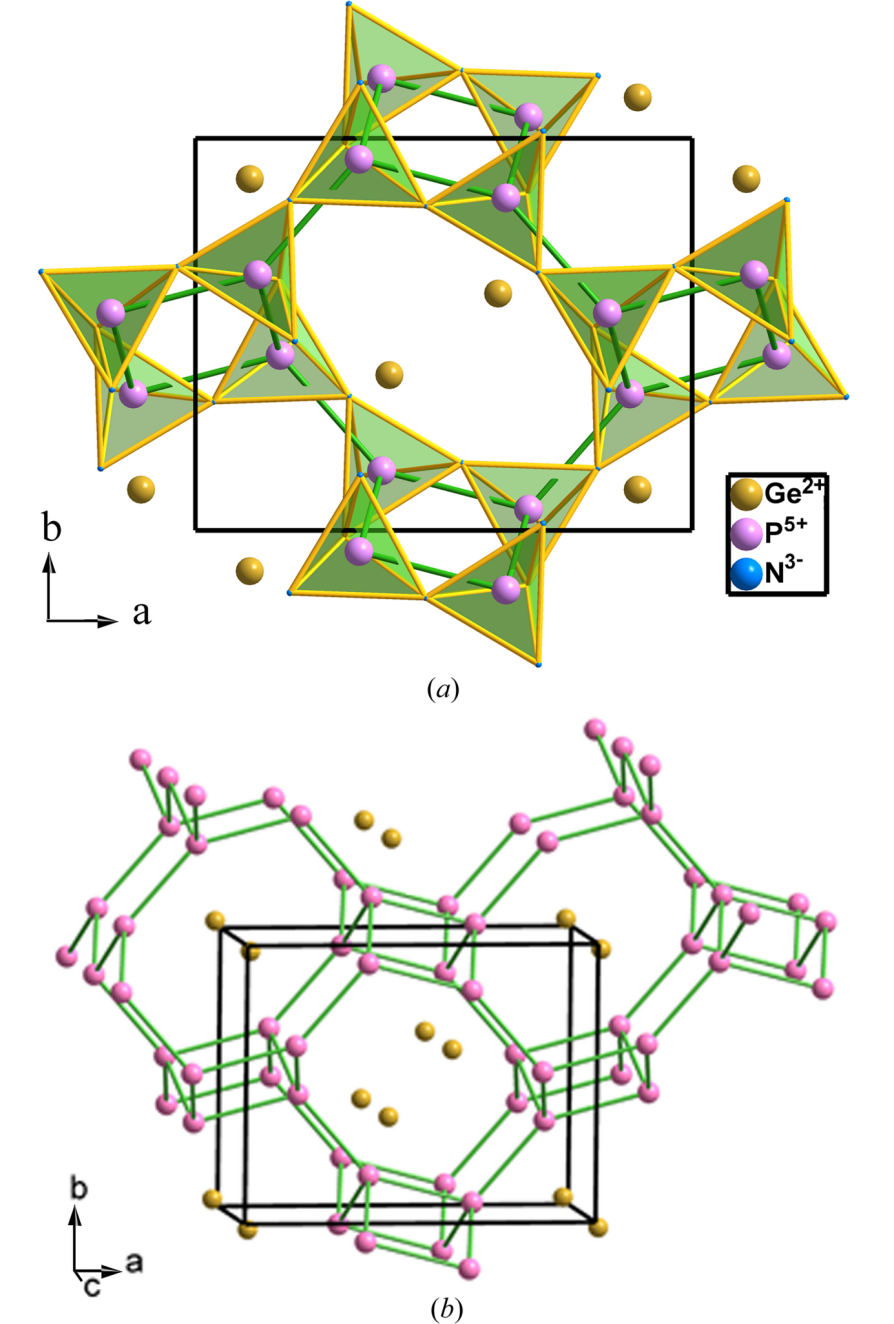
(*a*) View of the tetrahedral PN_4_ network of Ge^II^P_2_N_4_ along the *c* axis showing the octagonal tunnels lodging the Ge^II^ atoms. (*b*) The four-connected P skeleton (**sra** type) formed by the accordion-like ladders interconnected yo each other to build the octagonal tunnels.

The synthesis and crystal structures of Ge^II^P_2_N_4_ and Ge^IV^PN_3_ have been reported by Vogel *et al.* (2020 ▸), de Boer *et al.* (2023 ▸) and Ambach *et al.* (2024 ▸). The peculiarity of Ge^II^P_2_N_4_ resides in the presence of divalent Ge^II^ cations, which must preserve a nonbonding lone electron pair (LEP) that is localized on the 4*s* orbital (Ambach *et al.*, 2023 ▸). The orthorhombic (*Pna*2_1_, No. 33) structure of Ge^II^P_2_N_4_ is represented in Fig. 6 ▸ and can be rationalized by applying the EZKC: the Ge atom transfers two electrons to two of the four N atoms, and each P atom transfers one electron (two in total) to the other two N atoms, resulting in the pseudo-formula Ge^2+^(P^+^)_2_(N^−^)_4_ ≡ [Ψ-Zn][Ψ-SiO_2_]_2_.

**Figure 7:**
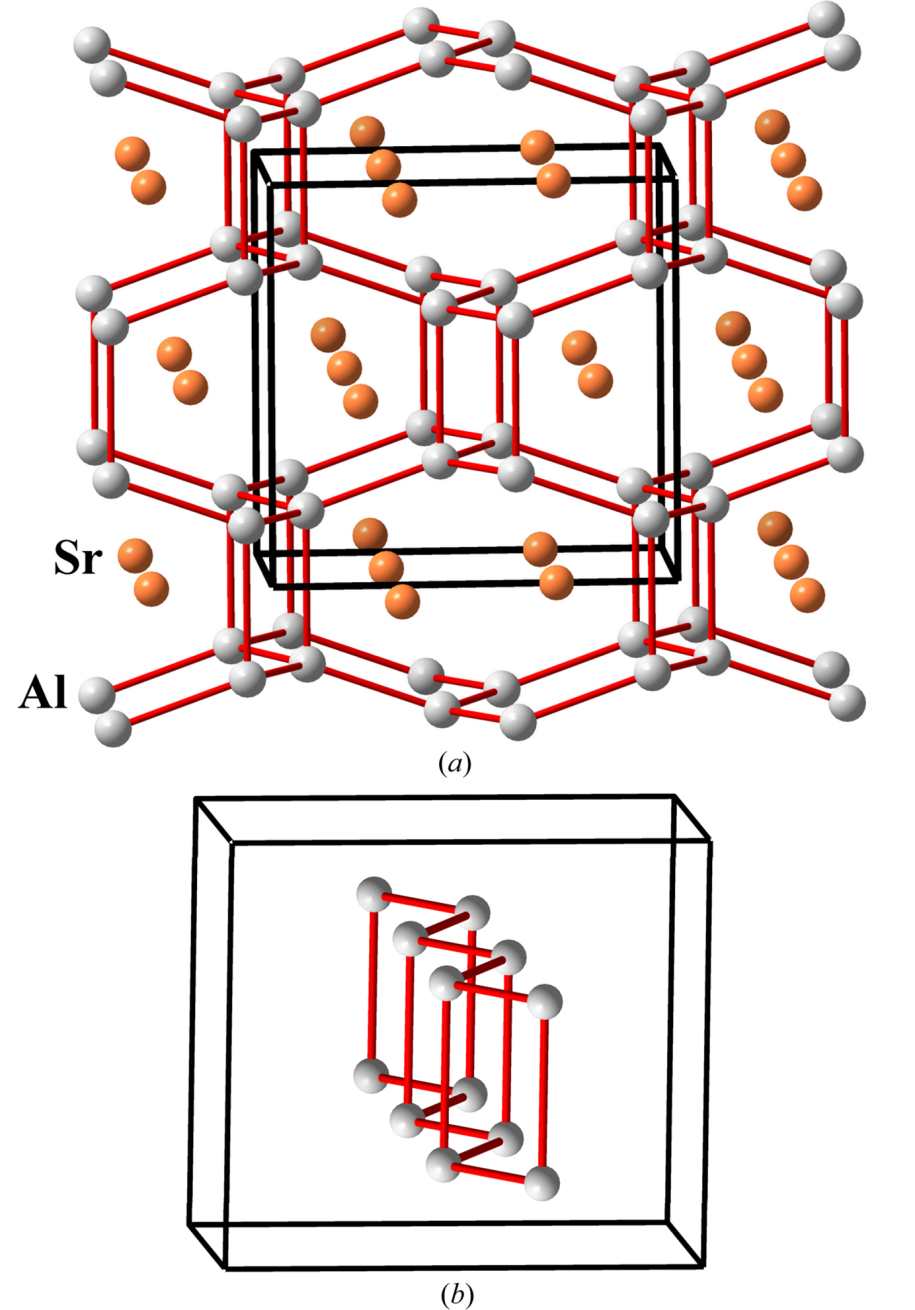
(*a*) Structure of the Zintl phase SrAl_2_ (**sra** type). Al atoms, converted into Ψ-Si, form a four-connected skeleton, in accordance with the 8-N rule. (*b*) One of the accordion-like moieties that are condensed in the structure of the Zintl polyanion Ψ-Si_2_. The same skeleton is formed by the [AlSi]^−^ ≡ Ψ-[Si_2_] subarray in isostructural RbAlSiO_4_.

In Ge^II^P_2_N_4_, like in other phospho­nitrides described in this article, the P atoms act as tetrels (Ψ-Si) so that the underlying P net is four-connected whose topology is of the **sra** type. The P atoms are coordinated tetrahedrally by four N atoms [see Fig. 6 ▸(*a*)]. When the N (Ψ-O) atoms are omitted, we obtain the skeleton drawn in Fig. 6 ▸(*b*) in which the four-connected P(Ψ-Si) skeleton is unveiled. It is formed by four- and eight-membered rings and shows strong similarities with the structure of the Zintl phase SrAl_2_ drawn in Fig. 7 ▸(*a*). It is worth noting that the four *d*(P–P) distances (2.84, 2.85, 2.92 and 2.95 Å; mean value of 2.89 Å) are in good agreement with the sum of the nonbonding radii for P atoms (2*R*_P_ = 2.92 Å) reported by O’Keeffe & Hyde (1981 ▸).

Curiously, the different structure of Ge^II^P_2_N_4_, when compared with those of the other *M*P_2_N_4_ (*M* = AE) compounds, has been interpreted as due to the location of LEPs on the Ge^II^ atoms (Vogel *et al.*, 2020 ▸). However, we consider that it can be interpreted otherwise. As we have mentioned above, the [Ψ-SiO_2_]_2_ framework is similar to that of the Al skeleton (Ψ-Si atoms) in the Zintl phase SrAl_2_ [Fig. 7 ▸(*a*)]. Since the Sr^2+^ cations (Ψ-Kr) do not have LEPs, this type of skeleton, found in Ge^II^P_2_N_4_, cannot be correlated with the presence of LEPs in Ge^II^ atoms. Instead, it should be regarded as a new example of the Ψ-Si skeleton that, in this case, results from the transfer of electrons from the less electronegative Ge and P atoms to the more electronegative N atoms, thus converting Ge and P atoms into Ψ-Zn and Ψ-Si atoms, respectively, and yielding the pseudo-formula [Ψ-Zn^0^][Ψ-SiO_2_]_2_.

**Figure 8:**
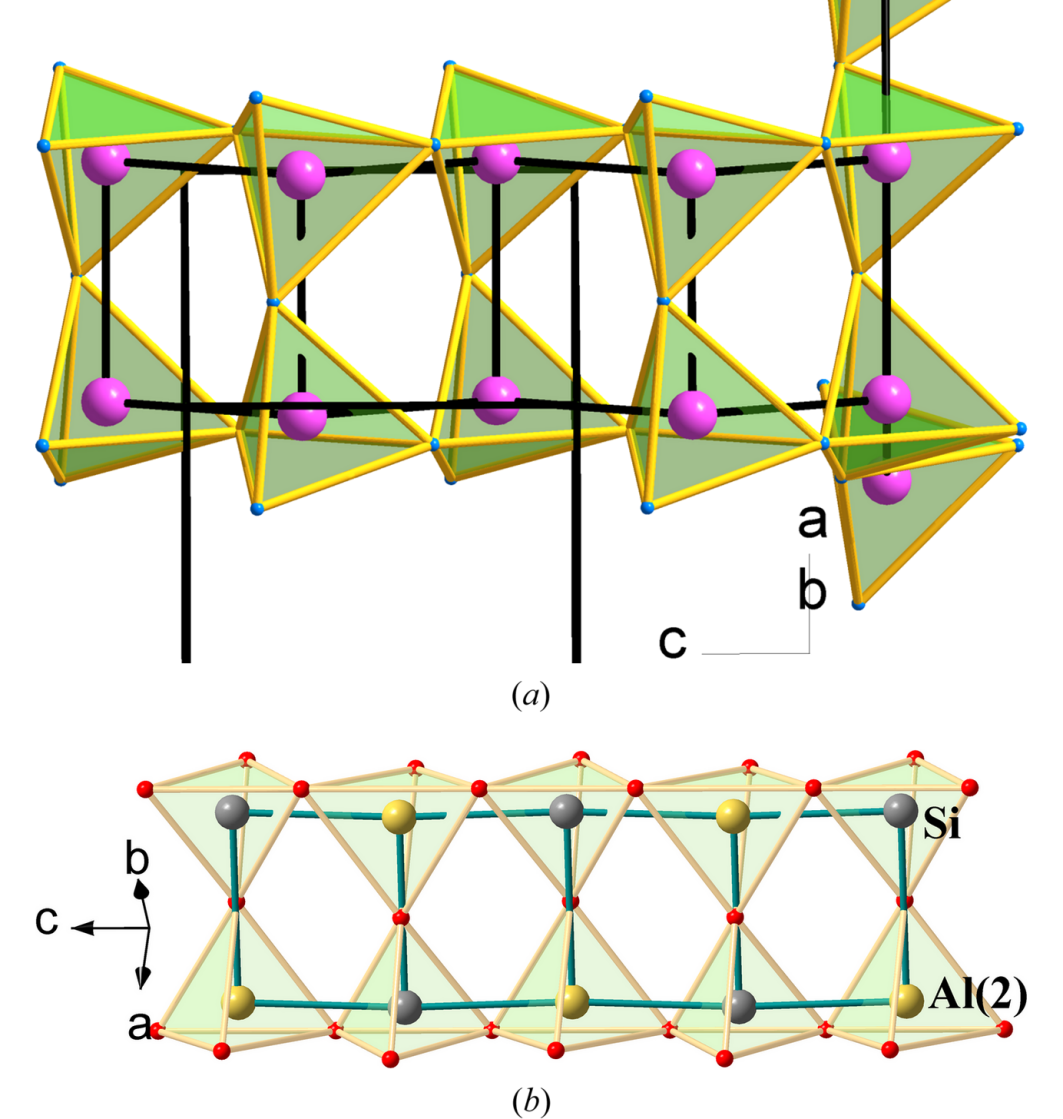
(*a*) One three-connected ladder-like fragment of PN_4_ tetrahedra extracted from Ge^2+^(P^+^)_2_(N^−^)_4_ ≡ Ψ-Zn^0^Si_2_O_4_ structure represented in Fig. 6 ▸(*a*). On the right-hand side, we have maintained two additional tetrahedra to illustrate its provenance from a four-connected network. P: violet; N: blue. (*b*) The partial structure of the [AlSiO_5_]^3−^ ≡ Ψ-P_2_O_5_ in Al_2_SiO_5_ (sillimanite). Al: yellow; Si: dark gray; O: red.

Alternatively, the structure can be rationalized as a condensation of the accordion-like ladders, like that represented as a separated moiety in Fig. 7 ▸(*b*). In these ladders, each atom (P atoms in Ge^II^P_2_N_4_ and Ψ-Si atoms in SrAl_2_) is three-connected [see Figs. 6 ▸(*b*) and 7 ▸(*b*)] and becomes four-connected when the ladders condense in the 3D skeletons [see Figs. 6 ▸(*a*) and 7 ▸(*a*)]. The ladder, separated from the Ψ-Si skeleton of SrAl_2_ [Fig. 7 ▸(*b*)], has its analog extracted from the Ge^II^P_2_N_4_ structure in Fig. 6 ▸(*a*) and is separated in Fig. 8 ▸(*a*).

According to the 8-N rule, the three-connected motif, characteristic of pentels, is also finely tuned in sillimanite, one of the polymorphs of Al_2_SiO_5_, whose structure is represented in Fig. 8 ▸(*b*). The sillimanite structure has already been rationalized in terms of the EZKC (Santamaría-Pérez *et al.*, 2005 ▸). In particular, the three-connected Al and Si atoms in this structure can be understood if one of the two Al atoms, in this case Al1, acts as a donor and transfers two electrons to Al2 and the third valence electron to the Si atom. Consequently, both species, Al2 and Si, convert into Ψ-P yielding the pseudo-formula Al^3+^[Ψ-P_2_O_5_][Ψ-Ne][Ψ-P_2_O_5_].

As reported by Santamaría-Pérez & Vegas (2003 ▸) and Vegas (2018 ▸), and according to both the EZKC and the 8-N rule, the Ψ-P atoms should be three-connected. In the skeleton of the pseudo-formula [Ψ-Ne][Ψ-P_2_O_5_], the five O atoms are located near the Ψ-(P—P) bonds and on the LEP associated with each P atom, as it is clearly seen in the two structures shown in Fig. 8 ▸. It should be added that these two ladder-like substructures, P_2_O_5_ and [AlSiO_5_]^3−^, differ in that the one in sillimanite is almost planar [Fig. 8 ▸(*b*)], in contrast to the accordion-like configuration of the same motif in Ge^2+^(P^+^)_2_(N^−^)_4_ ≡ Ψ-Zn^0^Si_2_O_4_ [Fig. 8 ▸(*a*)].

The above explanation of the structure of GeP_2_N_4_ in terms of the EZKC can be extended to the isostructural compounds KAlSiO_4_ and KZnPO_4_. In the former, the transfer of one electron from K → Al, converts the Al atom into Ψ-Si which, with the real Si atom, forms a four-connected network of stoichiometry Si_2_O_4_ in the pseudo-formula [Ψ-Ar][Ψ-SiO_2_]_2_. In KZnPO_4_, the transfer of one electron from K → Zn, results in the pseudo-formula [Ψ-Ar][Ψ-AlPO_4_], whose AlP substructure forms a four-connected network as it does the binary AlP compound itself. Note also that AlPO_4_ is a SiO_2_ homeotype. In particular, the mineral berlinite, α-AlPO_4_ (*P*3_1_21, No. 152), is related to the α-quartz structure (with AlO_4_ and PO_4_ units replacing two SiO_4_ units), with its *c* axis being double that of quartz.

The structural significance of these Ge-containing compounds (Ge^IV^PN_3_ and Ge^II^P_2_N_4_) resides in that both structures are explained in the frame of the EZKC despite the different valence states of the Ge atoms. It is also worth mentioning that the donor Ge^IV^ atoms are octahedrally coordinated, a feature coincident with the Al^[6]^ and Si^[6]^ atoms that also act as donors in some aluminates and silicates, *e.g.* the octahedral coordination of the donor Al1 atom in sillimanite (Al_2_SiO_5_) (Vegas, 2018 ▸) and the Si^[6]^ atoms that coexist with Si^[4]^ atoms in the oxonitridosilicate Ce_16_Si_15_O_6_N_32_ (Köllisch & Schnick, 1999 ▸). This structure was reinterpreted by Liebau (1999 ▸). The double coordination of Si atoms was later interpreted by Santamaría-Pérez *et al.* (2005 ▸) in the context of the EZKC. The octahedral coordination of the Si^[6]^ atoms was attributed to their donor character (Si^4+^) whilst the tetrahedral coordination was assigned to the acceptor Si atoms (Si^*n*−^). It should be remarked that both Ge^[4]^O_4_ tetrahedra and Ge^[6]^O_6_ octahedra also coexist in the germanate (NH_4_)_2_Ge^[6]^[Ge^[4]^_6_O_15_] (Cascales *et al.*, 1998 ▸). This feature, earlier considered a rarity, was further rationalized by Vegas & Jenkins (2017 ▸) in terms of the EZKC. Because Ce_16_Si_15_O_6_N_32_ and (NH_4_)_2_Ge^[6]^[Ge^[4]^_6_O_15_] were obtained at ambient pressure, they break the idea that octahedral coordination for silicon and germanium can only occur under HP conditions, as in stishovite, the HP-SiO_2_ polymorph.

**Figure 9:**
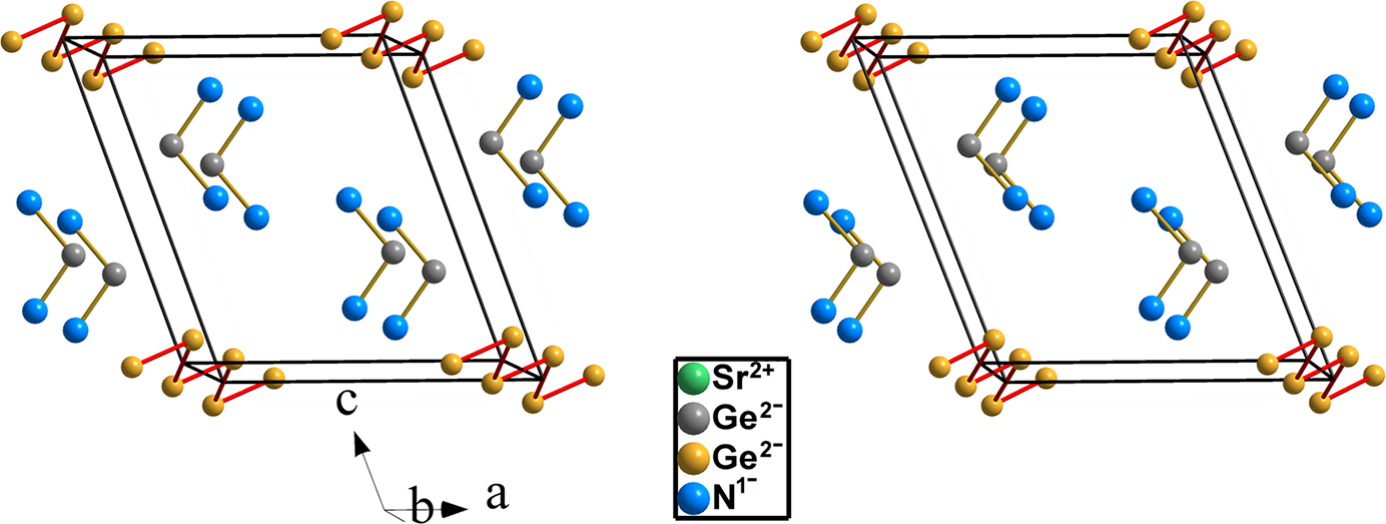
Stereopair of the unit cell of Sr_3_Ge_2_N_4_ showing the zigzag chains formed by the Ge^2−^ anions in the form of an extended Ψ-Se Zintl polyanion. The second Ge atom (gray spheres) also converted into Ψ-Se together with the N atoms (blue spheres), converted into Ψ-O, form molecules of Ψ-SeO_2_, similar with those occurring in real SO_2_. The Sr atoms have been omitted.

Thus, an important feature unnoticed by Ambach *et al.* (2024 ▸) is that the extended Zintl ion [Ge^2−^]_∞_ chains existing in the nitride Sr_3_Ge_2_N_2_, as well as the zigzag chains of P atoms underlying in the [PN_3_]^4−^ tetrahedral chains in Ge^IV^PN_3_, are the result of a similar electron transfer predicted by the EZKC. In both cases, the Ge and P atoms are converted into Ψ-Se and Ψ-S, respectively. The example of Sr_3_Ge_2_N_2_ is shown in Fig. 9 ▸.

### 2.4. CaP_2_N_4_ and SrP_2_N_4_ (hexagonal, *P*6_3_, No. 173)

**Figure 10:**
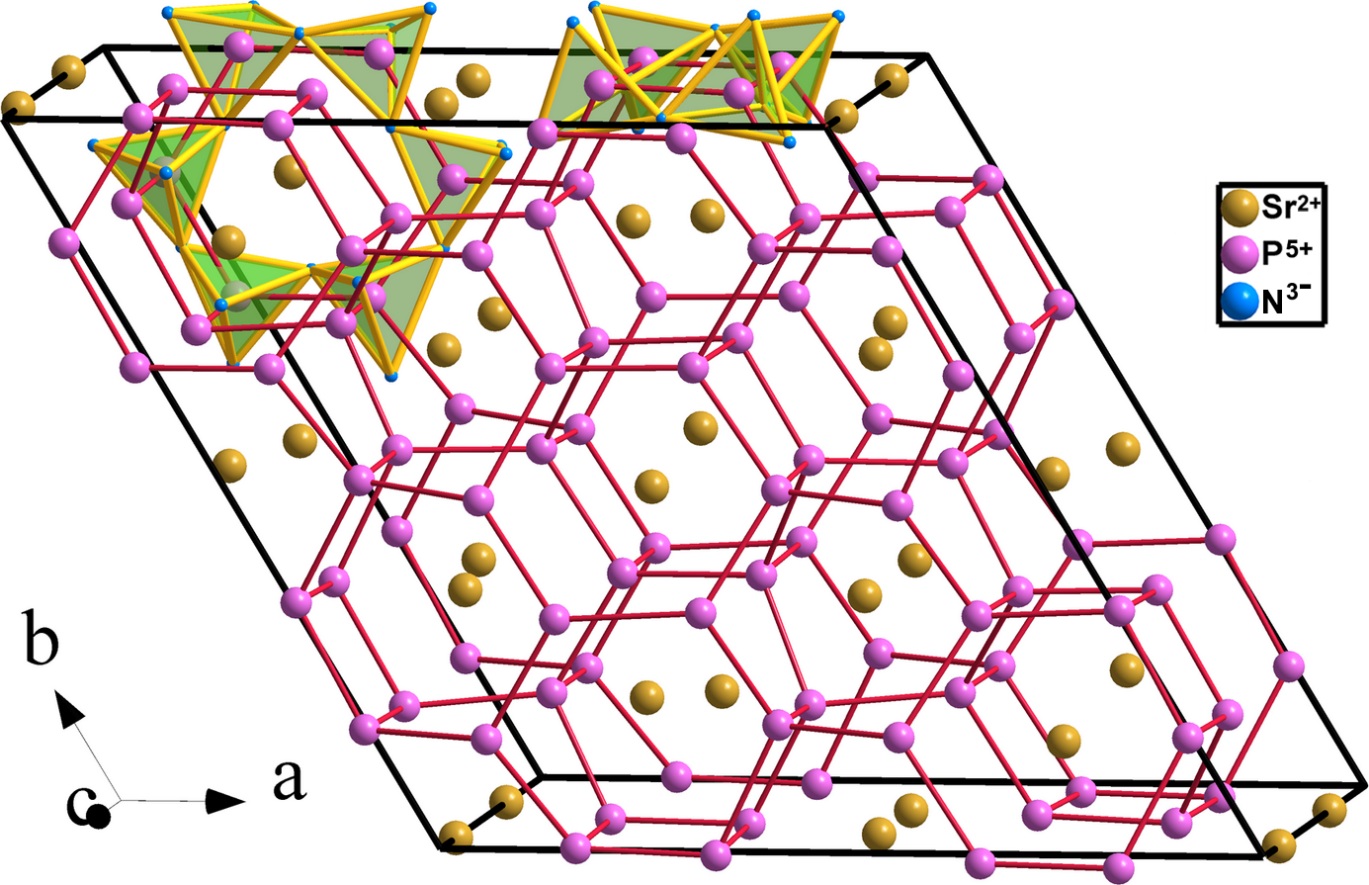
The four-connected P skeleton, connected with red lines, in compounds CaP_2_N_4_ and SrP_2_N_4_. It contains four-, six-, eight- and ten-membered rings. Some of them are drawn with their PN_4_ tetrahedra.

The syntheses and crystal structures of isostructural CaP_2_N_4_ and SrP_2_N_4_ were reported by Karau *et al.* (2007 ▸) and Pucher *et al.* (2015 ▸), respectively. The structure is hexagonal and is represented in Fig. 10 ▸. Both KAlSiO_4_ (megakalsilite) and KZnPO_4_ also belong to this structure type. Like the Be- and Ba-containing compounds, the P skeleton, drawn with red lines in Fig. 10 ▸, is four-connected, building a network of PN_4_ corner-sharing tetrahedra. The underlying P net is of the **tpd** type, adopted by the [AlGeO_4_] ≡ [Ψ-SiGeO_4_] partial structure in KAlGeO_4_. It is also reported as a hypothetical zeolite (PCOD8128676) in *TopCryst*. Sr atoms are located inside the hexagonal tunnels which run parallel to the *c* axis.

The four-connected P skeleton is represented in Fig. 10 ▸, projected near the *ab* plane, manifesting the puckered hexagonal **6^3^** layers, characteristic of the diamond and Si structures. However, these layers connect each other differently to those of Si. The diamond-like structure of Si only contains six-membered rings, while in CaP_2_N_4_ and SrP_2_N_4_, the interlayer connections yield four-, six-, eight- and ten-membered rings (see Fig. 10 ▸). Nevertheless, the important point is that each P atom is connected to four alike atoms. According to previous experience in the interpretation of the structures of aluminates and silicates in the context of the EZKC (Santamaría-Pérez & Vegas, 2003 ▸; Santamaría-Pérez *et al.*, 2005 ▸), this four-connectivity indicates that the P atom behaves as a tetrel (Ψ-Si).

This four-connectivity of P atoms can be explained with the EZKC if we admit that the Ca (Sr) atom transfers its two valence electrons to two N atoms and each P atom transfers one electron to the other two N atoms. This implies that Ca (Sr) becomes a Ψ-Ar (Ψ-Kr), P becomes a Ψ-Si, and N becomes a Ψ-O, so the pseudo-formula of CaP_2_N_4_ is [Ψ-Ar][Ψ-SiO_2_]_2_ and of SrP_2_N_4_ is [Ψ-Kr][Ψ-SiO_2_]_2_. Like in the structures of silica and in the skeletons of silicates as well, the N atoms (Ψ-O) situate near the midpoint of each (Ψ-Si)–(Ψ-Si) contact, so forming the tetrahedral P_2_N_4_ (Ψ-Si_2_O_4_) network represented in Fig. 10 ▸.

### 2.5. BaP_2_N_4_ (cubic, *Pa*3, No. 205)

The cubic structure of BaP_2_N_4_ consists of Ba^2+^ cations embedded in a three-dimensional framework of corner-sharing PN_4_ tetrahedra, as already recognized by Karau & Schnick (2005 ▸). They stated that ‘from a formula point of view, the [P_2_N_4_]^2−^ substructure is isoelectronic with SiO_2_. However, its topology is quite different from any known SiO_2_ polymorph’. In this regard, we consider that this assertion, being correct, falls short in identifying the structural similarity of BaP_2_N_4_ with one of the HP phases of CaB_2_O_4_(VI) reported by Marezio *et al.* (1969 ▸) and the implications of this similarity.

**Figure 11:**
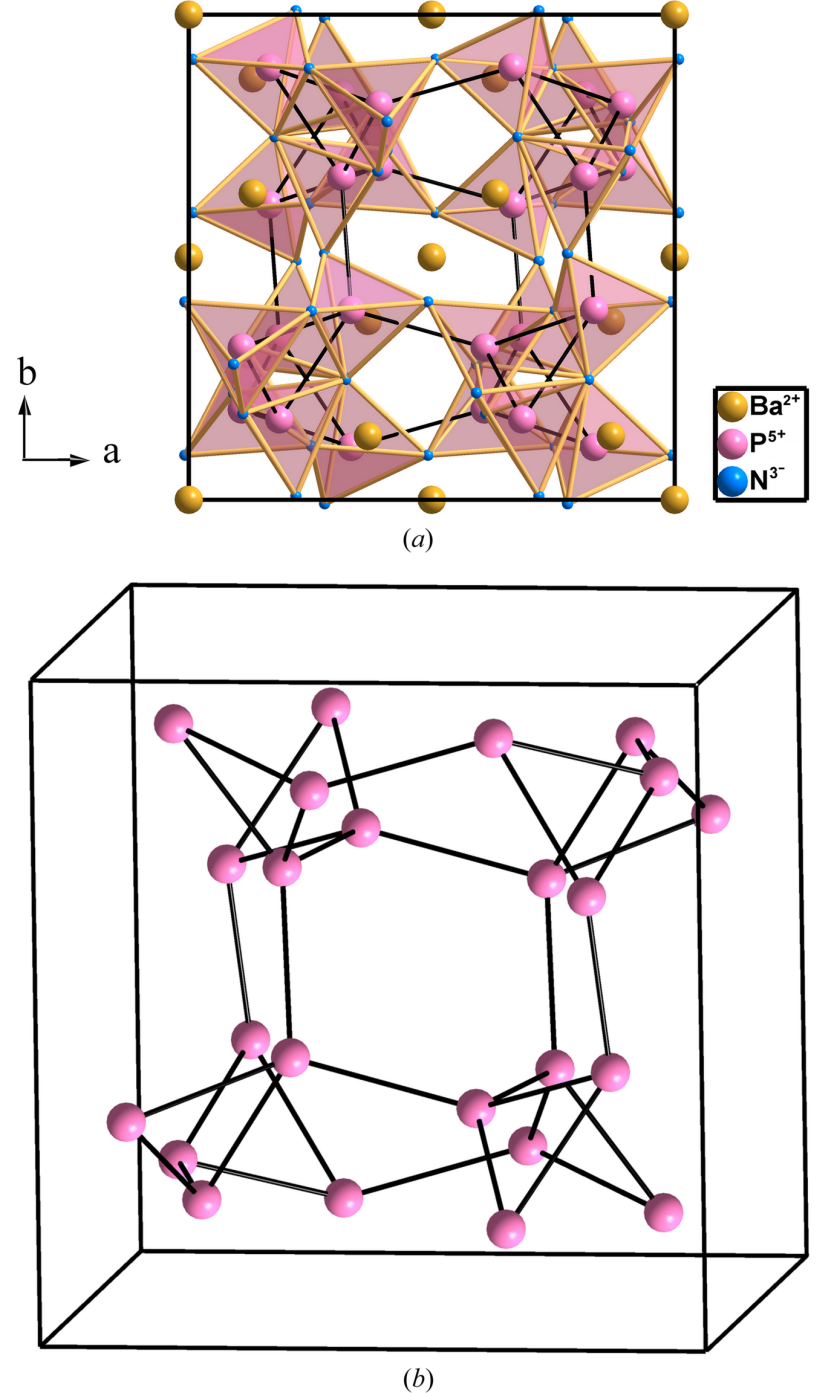
(*a*) Structure of BaP_2_N_4_ projected on the *ab* plane. The PN_4_ network forms hexagonal tunnels where the Ba atoms are lodged. (*b*) The P skeleton of BaP_2_N_4_ (**cbo**) shows a four-connectivity characteristic of tetrels. This can be understood with the EZKC if we consider that P atoms act structurally as Ψ-Si atoms.

The underlying net in BaP_2_N_4_ is **cbo** (from CaB_2_O_4_), also classified as hypothetical zeolite (PCOD8330894) in *TopCryst*. The structure contains a 3D network of PN_4_ tetrahedra sharing corners [Fig. 11 ▸(*a*)]. This network forms hexagonal tunnels where the Ba atoms are lodged. When the Ba and N atoms are neglected, the underlying four-connected P skeleton (Ψ-Si) is unveiled. This distorted P skeleton is drawn in Fig. 11 ▸(*b*) showing that each P atom is connected to four alike atoms, a feature that is characteristic of tetrels but not expected for pentels, *i.e.* the P atoms existing in BaP_2_N_4_. The fourfold connectivity is also not expected for triels (elements of Group 13) either. However, it is formed by the B atoms in CaB_2_O_4_. Next, we will show that the four-connected networks of both P and B atoms in BaP_2_N_4_ and CaB_2_O_4_(VI), respectively, can be rationalized with the EKZC.

The structure of CaB_2_O_4_(VI) can be understood if the two valence electrons of the Ca atom are transferred to the two B atoms to form Ψ-C atoms, so the compound can be formulated as Ca^2+^(B^−^)_2_O_4_ ≡ Ca^2+^[Ψ-CO_2_]_2_ ≡ [Ψ-Ar][Ψ-CO_2_]_2_. Similarly, considering that in BaP_2_N_4_ each Ba atom transfers its two valence electrons to two N atoms and that each P atom transfers one electron to the remaining two N atoms, we obtain the pseudo-formula unit Ba^2+^(P^+^)_2_(N^−^)_4_ ≡ Ba^2+^[Ψ-Si_2_O_4_] ≡ [Ψ-Xe][Ψ-SiO_2_]_2_. Both pseudo-formula units in CaB_2_O_4_(IV) and BaP_2_N_4_ are consistent with the four-connectivity observed for P in BaP_2_N_4_ and for B in CaB_2_O_4_(IV), as it occurs in the SiO_2_ polymorphs (Marezio *et al.*, 1969 ▸), since P and B atoms behave as pseudo atoms in Group 14.

### 2.6. BeP_2_N_4_ (rhombohedral, *R*3, No. 148 and cubic, *Fd*3*m*, No. 227)

**Figure 12:**
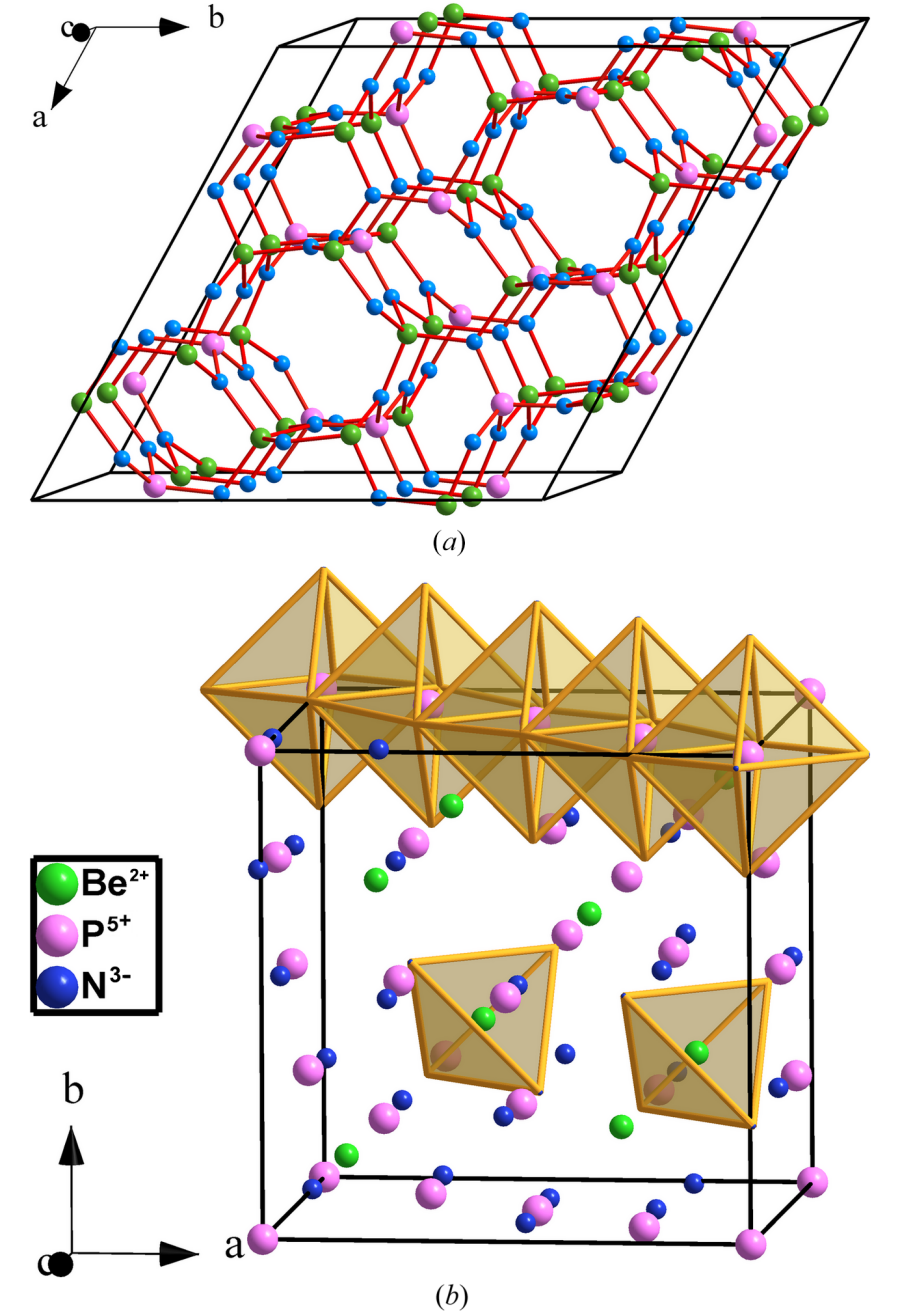
Structures of (*a*) phe-BeP_2_N_4_ and (*b*) sp-BeP_2_N_4_. In (*a*) both Be and P atoms are tetrahedrally coordinated. In (*b*) the P atoms center PN_6_ octahedra whilst the Be atoms center BeN_4_ tetrahedra.

The phe-BeP_2_N_4_ phase (*R*3, No. 148) is of the **lcs** topological type. It was synthesized according to 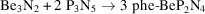
at high-pressure and high-temperature conditions (Vogel *et al.*, 2020 ▸).

phe-BeP_2_N_4_ is isostructural to mineral phenakite (Be_2_SiO_4_) although Be plays the role of Si and P the role of Be. phe-BeP_2_N_4_ transforms into a spinel-type structure (hereafter sp-BeP_2_N_4_) at 47 GPa and 1800 K. Both phenakite and spinel structures are drawn in Fig. 12 ▸. The spinel phase is quenchable to room pressure yielding an ultra-incompressible material (Vogel *et al.*, 2019 ▸). Vogel and coworkers stated that this new phase was predicted from theoretical calculations. However, they focused on the spinel-type structure and did not discuss other possible intermediate phases, like the olivine-type structure, in the reported phenakite → spinel transition. It should be recalled that the olivine structure is typically a phase observed at lower pressures than the spinel phase in many *M*_2_*X*O_4_ compounds and that the olivine → spinel transition at HP is undergone by several *M*_2_*X*O_4_ compounds (Vegas *et al.*, 2009 ▸; Vegas, 2018 ▸). The paradigmatic example is the olivine → spinel transition undergone by mineral forsterite (Mg_2_SiO_4_) at 22 GPa and 1273 K (Sasaki *et al.*, 1982 ▸). This point is important because, unlike phenakite (Be_2_SiO_4_) in which both Be and Si atoms are tetrahedrally coordinated (P and Be atoms in BeP_2_N_4_), in both the olivine and spinel structures, the Mg atoms are octahedrally coordinated and Si atoms center the SiO_4_ tetrahedra [see Fig. 12 ▸(*b*)].

The description of the phenakite-like structure of BeP_2_N_4_ [see Fig. 12 ▸(*a*)] is not an easy task even applying the EZKC that has allowed us to describe the previous phospho­nitrides. Our first approach was to investigate whether the BeP_2_ substructure in BeP_2_N_4_ (the Be_2_Si substructure in Be_2_SiO_4_) had any structural similarity with any of the binary alloy structures, according to the idea of O’Keeffe & Hyde (1985 ▸) of describing the structures of oxides as oxygen-stuffed alloys. A topological analysis carried out with the *ToposPro* (Blatov *et al.*, 2014 ▸) package revealed that no binary alloy structure matched those of the BeP_2_ or Be_2_Si subarrays in BeP_2_N_4_ and Be_2_SiO_4_, respectively. This issue confers a great singularity to the phenakite-type structure which could be an indication of a very narrow range of stability for this unusual skeleton. Only ten isostructural compounds have been collected in the ICSD: Be_2_SiO_4_, Li_2_BeF_4_, Li_2_SeO_4_, Zn_2_SiO_4_, Li_2_WO_4_, LiAlSiO_4_, LiAlGeO_4_, β-Si_3_N_4_, LiGaSiO_4_ and LiGaGeO_4_.

**Figure 13:**
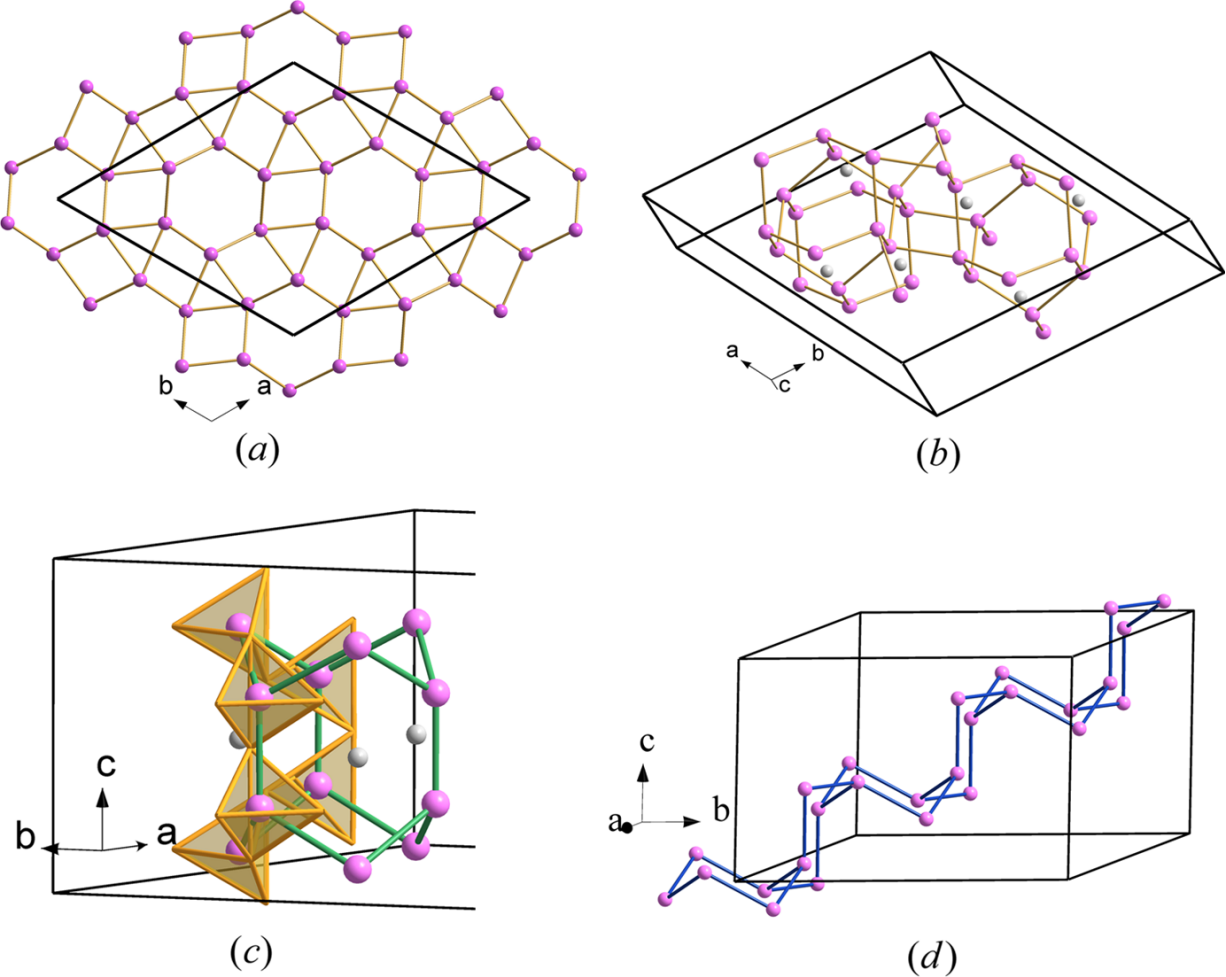
(*a*) The P substructure (violet spheres) in phe-BeP_2_N_4_ projected on the *ab* plane. (*b*) Perspective view of two connected hexagonal tunnels formed by the P atoms in BeP_2_N_4_, where some Be atoms (gray spheres) have been inserted for reference. The fourfold connectivity of the P skeleton is clearly evident. (*c*) A fragment of the lonsdaleite-type tunnel formed by the P atoms in phe-BeP_2_N_4_ which shows the tetrahedral coordination of the P atoms. PN_4_ tetrahedra are drawn in one of the rings of boat conformation. The linear -P-Be-P-Be-P- chains, parallel to the *c* axis, are clearly evident. (*d*) A fragment with the diamond structure which connects the tunnels with the lonsdaleite structure. P: violet; Be: gray.

**Figure 14:**
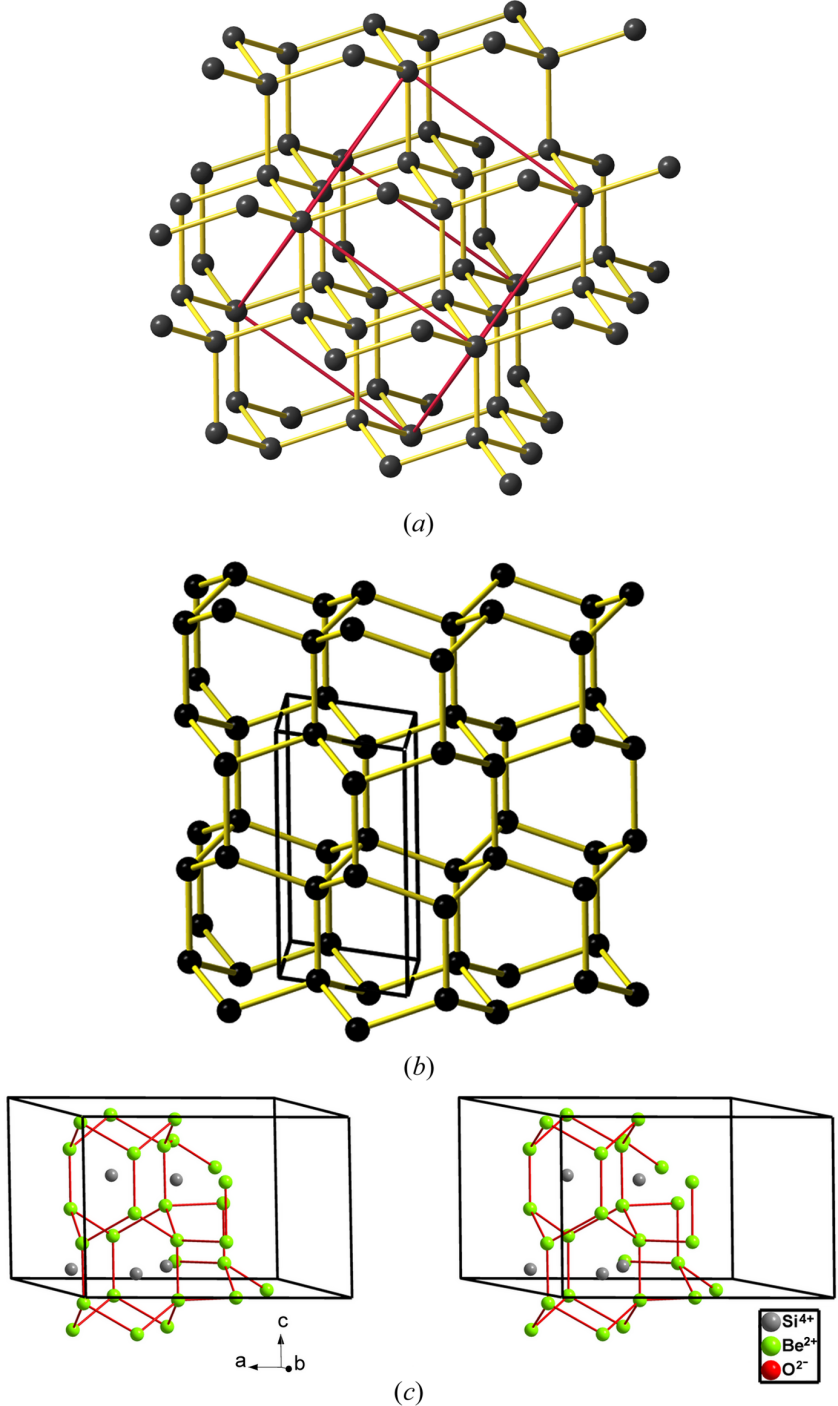
(*a*) Perspective view of the tetrahedral diamond structure (**dia**) showing the chair conformation rings. (*b*) The structure of hexagonal diamond (**lon**) showing the horizontal layers of rings in chair conformation and the vertical layers of hexagons in boat conformation. (*c*) Stereopair in perspective of the BeP_2_ substructure in phe-BeP_2_N_4_ showing two interconnected lonsdaleite-type tunnels yielding the irregular four-connected P skeleton. The two conformations (boat and chair) of the six-membered rings are visible. Be: gray; P: violet.

The phenakite structure of BeP_2_N_4_ will be described based on the partial structure of the P atoms. Its singularity resides in that the P atoms form a three-dimensional four-connected skeleton which has been drawn, in projection, in Fig. 13 ▸(*a*). When drawn in perspective [Fig. 13 ▸(*b*)], one can see that the P skeleton contains extended hexagonal tunnels running parallel to the *c* axis and centered at (*x*, *y*) positions (0, 0), (⅓, ⅔) and (⅔, ⅓) in the *ab* plane [see also Fig. 12 ▸(*a*)]. These tunnels are blocks of a lonsdaleite-type structure (also wurtzite type), containing both chair and boat conformation six-membered rings. A fragment of such a tunnel is drawn in Fig. 13 ▸(*c*) to show the chair-conformed rings forming the layers perpendicular to the *c* axis. These layers are stacked in an …*ABABAB*… sequence which gives rise to additional boat-conformed rings, running parallel to the *c* axis [see also Fig. 14 ▸(*c*)].

The relative positions of these tunnels are such that, when they connect to each other, new six-membered rings are formed, all of them having a chair conformation like in the diamond-type structure. The result is the irregular tetrahedral skeleton represented in Fig. 14 ▸(*c*). Thus, the P skeleton in BeP_2_ is formed by lonsdaleite-type blocks interconnected by diamond-type fragments like that represented in Fig. 13 ▸(*d*). Both fragments, **lon** type [Fig. 13 ▸(*c*)] and **dia** type [Fig. 13 ▸(*d*)] can be compared with the respective structures of silicon in Figs. 14 ▸(*a*) and 14 ▸(*b*). The Be atoms, located midway between the two opposite P atoms of the rings in boat conformation, give rise to extended linear chains -P-Be-P-Be-, parallel to the *c* axis, which can be seen in Figs. 13 ▸(*c*) and 14 ▸(*c*). In the former, the PN_4_ tetrahedra have been highlighted in a boat-conformed hexagon.

The four-connected skeleton formed by the P atoms in phe-BeP_2_N_4_ is unexpected. According to the 8-N rule, that connectivity is characteristic of tetrels (C, Si, Ge) but not for pentels. However, such P skeletons can be rationalized with the EZKC as follows. If we assume that the Be atom transfers its two valence electrons to two N atoms and that the two P atoms each transfer one electron to the two remaining N atoms, then the Be atom becomes Ψ-He, the two P atoms become Ψ-Si and the four N atoms become Ψ-O, so that BeP_2_N_4_ can be rewritten as Be^2+^(P^+^)_2_(N^−^)_4_ ≡ Be^2+^[Ψ-Si_2_O_4_] ≡ [Ψ-He][Ψ-SiO_2_]_2_, *i.e.* a new He-filled [Ψ-SiO_2_] structure intermediate between those of cristobalite and tridymite. Recall that the Si skeleton in SiO_2_ (tridymite) is of the lonsdaleite type.

This interpretation, based on the four-connected skeleton [Figs. 13 ▸(*b*) and 14 ▸(*c*)], provides a new example of the general trend of binary/ternary alloys to form four-connected skeletons (Vegas & García-Baonza, 2007 ▸). Since the N atoms are transformed into Ψ-O atoms, they are located near each (Ψ-Si)—(Ψ-Si) bond thus resulting in the tetrahedral coordination shown for the P atoms, as explained for other oxides such as aluminates and silicates (Santamaría-Pérez *et al.*, 2005 ▸). It should be added that if we allow only the electron transfer from Be → N and not that from P → N, then, the resulting pseudo-formula would be [Ψ-He][Ψ-PON]_2_ which corresponds to that of the real PON compound, isoelectronic with SiO_2_, obtained as quartz, cristobalite and moganite types of SiO_2_ (Léger *et al.*, 1999 ▸).

### 2.7. Alternative electron transfers for BeP_2_N_4_ compatible with the EZKC 

In addition to the already proposed application of the EZKC to explain the structure of phe-BeP_2_N_4_ as [Ψ-He][Ψ-SiO_2_]_2_, three alternative pseudo-formulae can be obtained, which could potentially help us to understand the phe-BeP_2_N_4_ structure. Note that all four different pseudo-formulae according to the EZKC result from the different possible distributions of the valence electrons between the three types of atoms. The difference between the pseudo-formula explained in Section 2.6 and the three interpretations quoted below is that the three new pseudo-formulae correspond to olivine-type pseudo-oxides.

The additional EZKC pseudo-formulae for phe-BeP_2_N_4_ are:

(i) Ψ-Al_2_BeO_4_ (chrysoberyl, olivine-type). If the P atoms transfer four electrons to the four N atoms, then the P atoms become Ψ-Al and the N atoms transform into Ψ-O.

(ii) Ψ-AlMg(Ψ-BO_4_) (pseudo-sinhalite, olivine-type). The two P atoms transfer a total of five electrons (one to Be and one each to four N atoms). Of the two P atoms, the one that transfers two electrons becomes Ψ-Al and the other that transfers one electron transforms into Ψ-Mg. On the other hand, the Be and N atoms become Ψ-B and (Ψ-O), respectively, yielding the Ψ-BO_4_ group.

(iii) Ψ-Mg_2_(CO_4_) (non-existing; analogous to the olivine-type Mg_2_SiO_4_). The P atoms can transfer six electrons (two to Be and one each to four N atoms). Thus, the two P atoms become Ψ-Mg, Be transforms into Ψ-C and the N atoms into Ψ-O, so that BeN_4_ can be reformulated as Ψ-CO_4_.

Note that Al_2_BeO_4_ also has its analog in the spinel Al_2_MgO_4_ (**spn**) and that the non-existing Mg_2_CO_4_ has its analog in forsterite Mg_2_SiO_4_.

**Figure 15:**
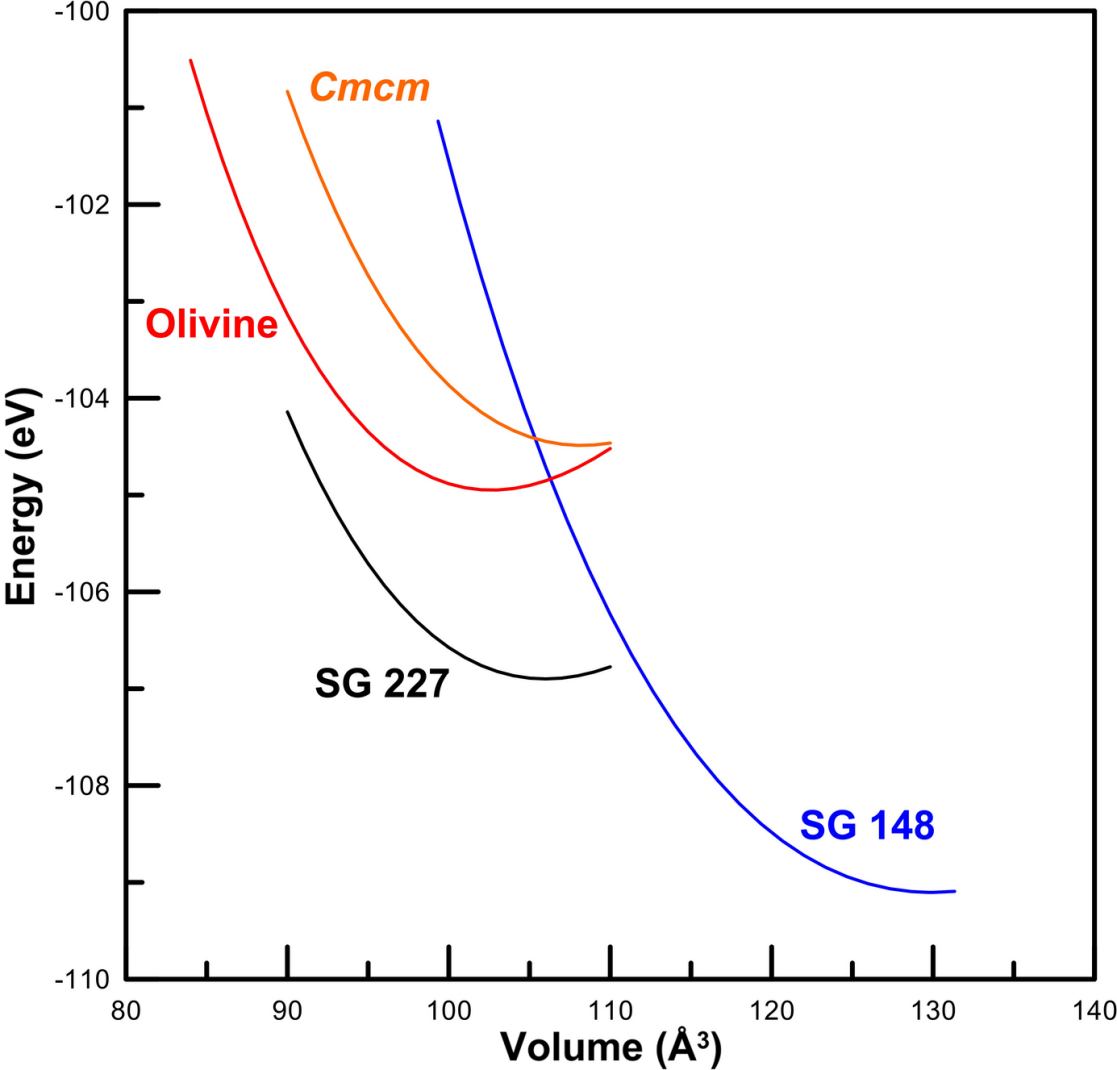
Energy as a function of volume for the phenakite-like (*P*3, No. 148), olivine-like (*Pnma*, No. 62), distorted-olivine-like (*Cmcm*, No. 63) and spinel-like (*Fd*3*m*, No. 227) structures in BeP_2_N_4_.

To prove whether these additional EZKC pseudo-formulae leading to olivine-like phases could be observed in BeP_2_N_4_ under any pressure range, for example, as an intermediate stage between the phenakite-like and spinel-like phases, we have performed *ab initio* calculations of the olivine structure in BeP_2_N_4_. We found that this phase is not thermodynamically stable between the phenakite-like and spinel-like structures. In addition, we have simulated a distorted olivine (*Cmcm*, No. 63) phase and found that it is not competitive with the phenakite and spinel phases (see Fig. 15 ▸). Therefore, BeP_2_N_4_ is a rather curious *AB*_2_*X*_4_ compound since it does not show an olivine-like phase at lower pressures than those found for the spinel-like phase. This means that the EZKC explanation we gave in Section 2.6 seems to be the most likely possibility to explain the phenakite structure in BeP_2_N_4_.

An alternative way of checking which of the three alternative pseudo-formulae of BeP_2_N_4_, compatible with the EZKC, is the most probable to explain the structure of phe-BeP_2_N_4_ is to make first-principles simulations of phe-BeP_2_N_4_ to obtain the atomic (B, P and N) charges with density-based and orbital-based methods. In this way, the Bader atomic charges have been calculated according to the density-based Quantum Theory of Atoms in Molecules (QTAIM) (Bader, 1985 ▸) using the *CRITIC2* software (Otero-de-la Roza *et al.*, 2009 ▸; Otero-de-la Roza *et al.*, 2014 ▸). In addition, Löwdin and Mulliken charges have been calculated using the orbital-based *LOBSTER* software (Nelson *et al.*, 2020 ▸).

For phe-BeP_2_N_4_, the average Bader charges are Be^+1.10^, P^+2.40^ and N^−1.48^. The average Löwdin charges are Be^+1.50^, P^+1.85^ and N^−1.30^. The average Mulliken charges are Be^+1.70^, P^+2.05^ and N^−1.45^. These results clearly demonstrate that Be and P atoms donate charge to N atoms; a result that agrees with the donor character of Ge and P atoms in Ge^IV^PN_3_. It can be observed that QTAIM calculations provide the most ionic picture as already discussed in the literature (Kaupp, 2014 ▸). In conclusion, the last three EZKC pseudo-formulae, which assume that Be does not donate charge or even that it accepts charge, can be ruled out to explain the structure of phe-BeP_2_N_4_. This result is consistent with the lack of observation of the olivine structure in BeP_2_N_4_ as a possible structure in the pressure range explored (see Fig. 15 ▸).

### 2.8. LiGaGe and LiGaGeO_4_

**Figure 16:**
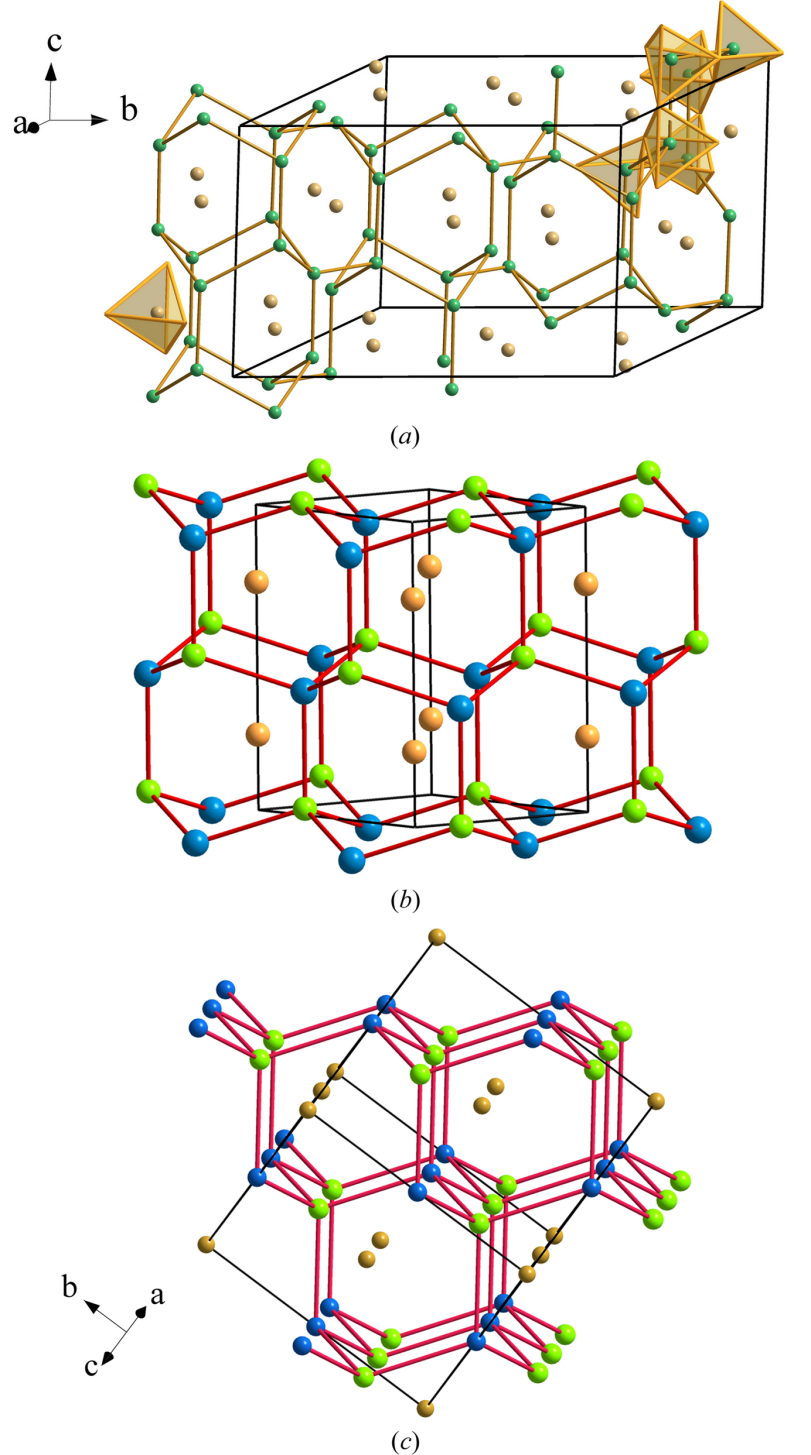
(*a*) Perspective view of the [GaGe]^−^ subarray [(Ψ-Ge)(Ge)] in phe-LiGaGeO_4_ (**lcs** type), which can be compared with the BeP_2_ substructure in phe-BeP_2_N_4_ represented in Fig. 13 ▸(*c*). The drawing shows the connected **lon**-type tunnels yielding the four-connected P skeleton. The two conformations (boat and chair) of the six-membered rings are visible. Be: gray; P: violet. (*b*) The filled wurtzite-type structure of the Zintl phase LiGaGe. The four-connected skeleton is built from the subarray [GaGe]^−^ ≡ Ψ-Ge. The Li atoms center the hexagonal tunnels running parallel to the *c* axis. Compare with Fig. 13 ▸(*b*). Li: ochre; Ga: green; Ge: dark blue. (*c*) The stuffed diamond-like structure of the half Heusler phases such as LiAlSi and LiGaSi.

We discussed in Section 2.6 that the cation subarray (BeP_2_) in phe-BeP_2_N_4_ does not match the structure of any other binary (ternary) alloy. This lack of reference has led us to search for possible relationships between the cation substructures of the isostructural phenakite-type compounds and the structures of their corresponding alloys. The analyzed compounds were those mentioned in Section 2.6 and for only one of them, LiGaGeO_4_, we have found the existence of a Zintl phase (LiGaGe) (**lon** type) with the same composition as the cation array of the oxide. The structure of LiGaGeO_4_ (**lcs** type) is represented in Fig. 16 ▸(*a*) and that of the Zintl phase LiGaGe is drawn in Fig. 16 ▸(*b*).

Both structures can be interpreted as if the Li atoms would donate their valence electron to the Ga atoms, converting them into Ψ-Ge, so that LiGaGe can be reformulated as Li^+^[GaGe]^−^ ≡ [Ψ-He][Ψ-Ge_2_] which is equivalent to an He-filled structure identical to the lonsdaleite (wurtzite) type structure of Si(Ge), represented in Fig. 14 ▸(*b*). In the same manner, the phenakite-type structure of LiGaGeO_4_ can be formulated as a [Ge_2_O_4_] skeleton, filled with Li^+^ cations [Fig. 16 ▸(*a*)].

The important point is that the LiGaGe alloy has a filled wurtzite-type structure that corresponds with one of the fragments (hexagonal tunnels) building the partial structure of phe-LiGaGeO_4_. According to the Zintl concept, LiGaGe can be reformulated as Li^+^[Ga^−^Ge] ≡ [Ψ-He][Ψ-Ge]Ge ≡ Ψ-(He)Ge_2_, *i.e.* a He-filled lonsdaleite-type structure of Ge atoms. It is worth noting that a tetrahedral skeleton characteristic of tetrels (C, Si, Ge) is here adopted by the P atoms (pentels), whose skeleton, containing fragments (columns) of a lonsdaleite-type structure, is again drawn in perspective in Fig. 16 ▸(*a*) for comparison with Fig. 14 ▸(*b*).

It is now appropriate to compare the structures of LiGaGe and phe-BeP_2_N_4_. The wurtzite-type tunnels formed by the P atoms in Fig. 13 ▸(*b*) show their similarity to the wurtzite structure of Fig. 14 ▸(*a*) but, at the same time, the differences become apparent. They can be summarized in two features: (i) As discussed above, in BeP_2_N_4_ the Be atoms no longer center the wurtzite tunnels. (ii) The periodicity of the tunnels in the *ab* plane [Fig. 14 ▸(*a*)] is broken in BeP_2_N_4_ and the connection between the tunnels is now achieved by rings in chair-conformation [see Fig. 13 ▸(*b*)].

The structural relationship between phe-BeP_2_N_4_ and LiGaGe has allowed the structure of phe-BeP_2_N_4_ to be interpreted not only in terms of the EZKC but also of the oxidation pressure concept. Such an explanation can be attained if we consider the oxide LiGaGeO_4_ (**lcs** type), one of the ten compounds having the phenakite structure, as mentioned in Section 2.6. In the structure of LiGaGeO_4_ represented in Fig. 16 ▸, the Ga and Ge atoms are not distinguished because they could not be differentiated in the crystal structure determination.

### 2.9. sp-BeP_2_N_4_ (**spn** type)

**Figure 17:**
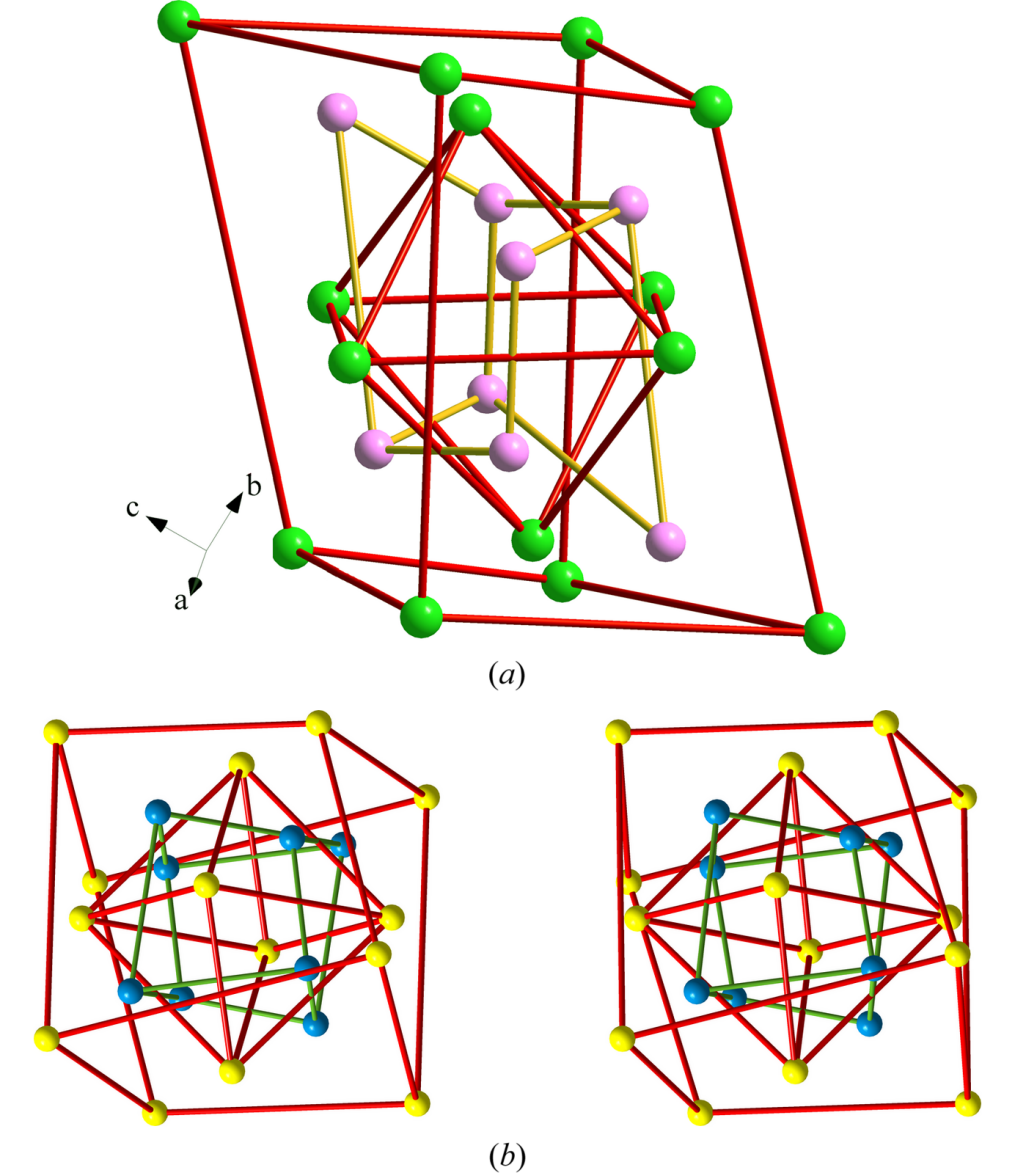
(*a*) Fragment of the hexagonal structure of phe-BeP_2_N_4_, isostructural to the mineral phenakite Be_2_SiO_4_. Be: green; P: violet (N atoms omitted). The fragment has similarities with the Li_2_S substructure of β-Li_2_SO_4_(*P*2_1_/*c*) at ambient conditions. S: yellow; Li: blue (O atoms have omitted). The stereopair of the room-temperature structure of β-Li_2_SO_4_ is shown in (*b*). The Li_2_S substructure forms a distorted antifluorite structure in which the S atoms form a distorted fcc array, with the 2*n* tetrahedral voids occupied by Li atoms. Note the irregularity of both the Li_8_ and S_8_ cubes, in contrast with the rather regular S_6_ octahedron. Reproduced from Vegas (2018 ▸) with permission.

We would also like to comment on the spinel-type phase of BeP_2_N_4_ [Fig. 12 ▸(*b*)]. It has been observed that the structure of phe-BeP_2_N_4_ contains slightly distorted octahedra of Be atoms (*i.e.* Si atoms in the mineral phenakite, Be_2_SiO_4_). The octahedra [Fig. 17 ▸(*a*)] are contained into an extremely distorted Be_8_ cube that contains not only the Be_6_ octahedron but also eight P atoms, drawn as purple spheres in Fig. 17 ▸(*a*). Despite its irregularity, the image in Fig. 17 ▸(*a*) is reminiscent of a fluorite-type structure, so that phe-BeP_2_N_4_ can be considered as a frustrated fluorite-type structure. This makes sense if we compare this structure with that of monoclinic β-Li_2_SO_4_(*P*2_1_/*c*) (Alcock *et al.*, 1973 ▸), shown in Fig. 17 ▸(*b*). The relationship between the structure of β-Li_2_SO_4_ and the antifluorite (anti-CaF_2_) structure was first noticed by Parfitt *et al.* (2005 ▸) and is much closer to the antifluorite-type than it is to phe-BeP_2_N_4_.

The similarity between both structures is only apparent because in the partial motif of phe-BeP_2_N_4_, represented in Fig. 17 ▸(*a*). The Be_6_ octahedra share faces, a feature that cannot exist in Li_2_SO_4_ which has a distorted fcc array of S atoms as it corresponds to its cubic fluorite-type structure.

Since the binary Li_2_S sulfide is fluorite type, the distortion of that cubic structure in the oxide Li_2_SiO_4_ has been explained by Vegas & Jenkins (2017 ▸) in the frame of the oxidation pressure concept. Thus, the insertion of four O atoms per Li_2_S unit provokes a strong distortion of the antifluorite (anti-CaF_2_) substructure of Li_2_S in Li_2_SO_4_, but when β-Li_2_SO_4_ is heated, the pressure exerted by the O atoms is released and a cubic phase with antifluorite structure is obtained. However, the pressure exerted by the O atoms is not high enough to cause the stabilization of any of the two possible HP phases of Li_2_S, *i.e.* anticotunnite or anti-Ni_2_In, as a substructure in Li_2_SO_4_. Nonetheless, when extra pressure is applied to β-Li_2_SO_4_, the olivine-type structure is obtained. For a complete analysis of the phase transitions observed in Li_2_S and Li_2_SO_4_, see Vegas (2018 ▸).

The inclusion of the Li_2_S/Li_2_SO_4_ structures pair into the discussion is appropriate, considering that one of the ten phenakite-type compounds is the closely related Li_2_SeO_4_ (Hartman, 1989 ▸). This is consistent with the rule that within a group of the Periodic Table of the Elements, the structure of a heavier analog (the selenate) can occur for the lighter one (the sulfate) at HP. Since the HP polymorph of Li_2_SO_4_ has an olivine-type structure, the existence of a phenakite structure, at lower pressures, should not be discarded. In the same way, the stabilization of an olivine-type structure for the selenate could also be expected.

**Figure 18:**
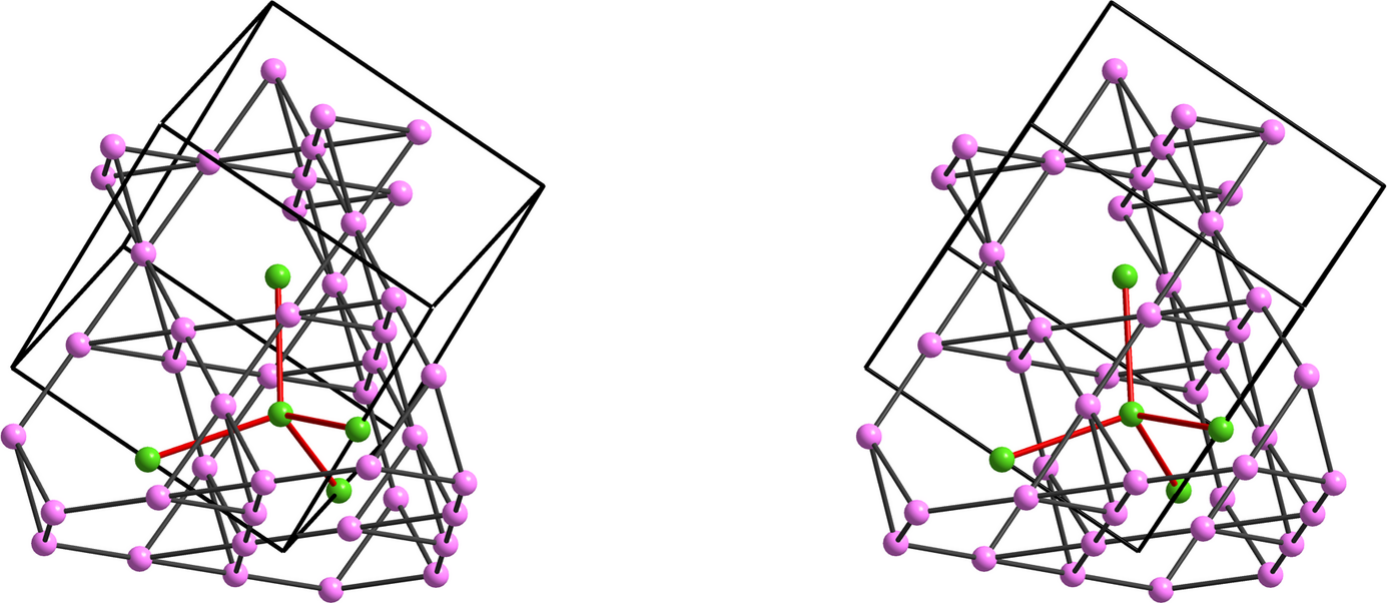
Stereopair of the MgCu_2_-type substructure formed by the BeP_2_ subarray in the HP spinel-type phase of BeP_2_N_4_. P: purple; Be: green (N atoms are omitted). The Be atoms center the P_12_ truncated tetrahedra. When the Be atoms are connected, they form a diamond-like network.

This reasoning can be also applied to the description of the sp-BeP_2_N_4_ structure. It is worth recalling that the connection of the structures of Li_2_S with those of the antifluorite and olivine types conveys with the transition path anti-CaF_2_ → anti-PbCl_2_ → anti-Ni_2_In → anti-MgCu_2_. This transition pathway, observed in several *AB*_2_ compounds (Vegas, 2011 ▸), also covers the phe → sp transition in BeP_2_N_4_ (Vogel *et al.*, 2020 ▸) because this transition involves the BeP_2_ → MgCu_2_ transition undergone by the cation substructures. Notice the partial antifluorite structure in Fig. 17 ▸(*a*) and the regular MgCu_2_-type of the BeP_2_ array drawn in Fig. 18 ▸.

It is important to note that both the alloy Be_2_C (the lighter analog of Be_2_Si) and Mg_2_Si exhibit an antifluorite-type structure, and that Mg_2_Si also adopts the MgCu_2_-type structure when the Si atoms are partially replaced by small amounts of Sn atoms (Boudemagh *et al.*, 2011 ▸). This agrees with the view of Vogel *et al.* (2020 ▸), who noticed that the Be atoms are at the center of BeN_4_ tetrahedra, whereas the P atoms are octahedrally coordinated (PN_6_ octahedra), like in the β-BP_3_N_6_ structure obtained at 47 GPa (Vogel *et al.*, 2019 ▸).

**Figure 19:**
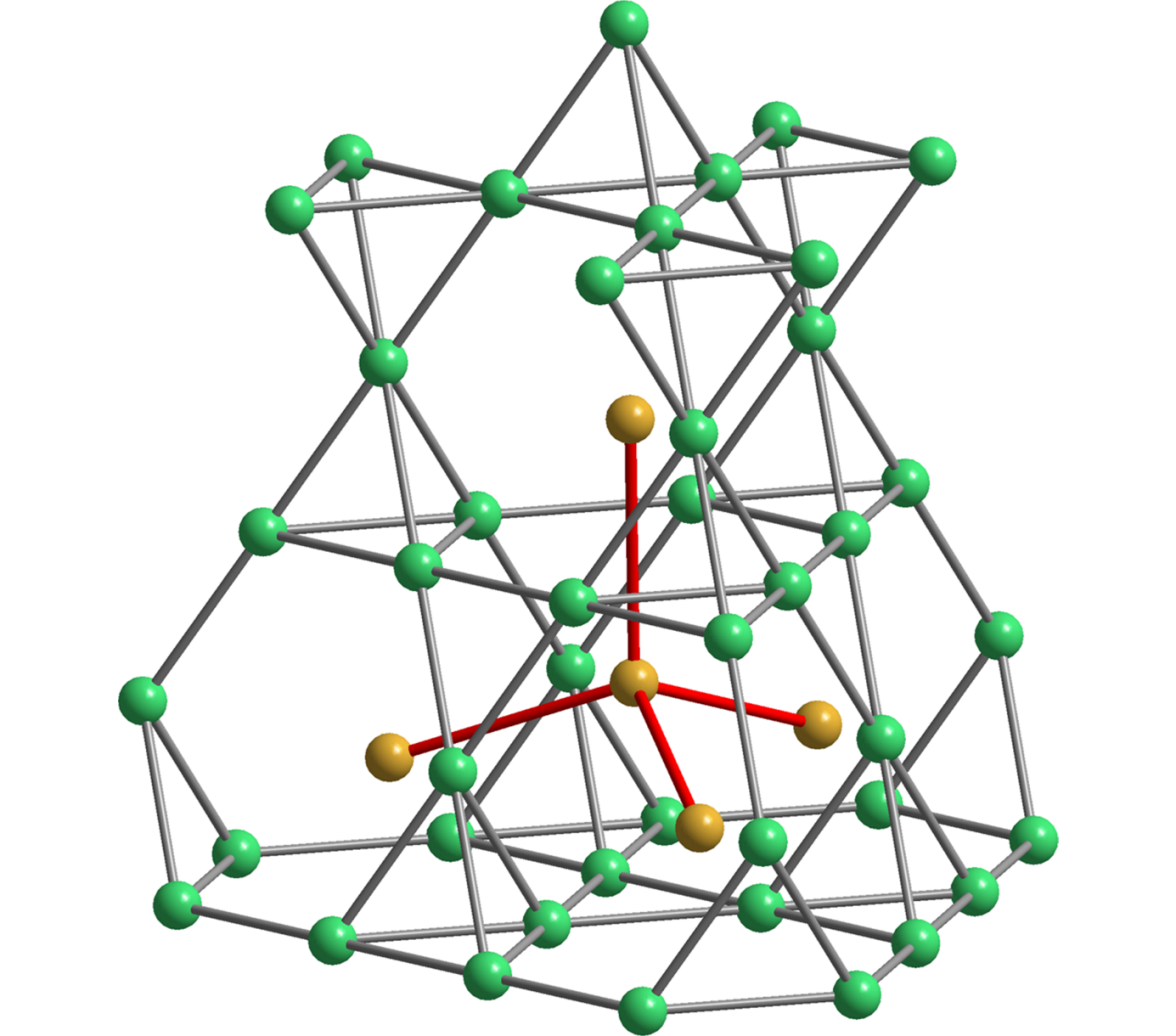
Structure of the cubic Laves phase MgCu_2_, identical to the cation subarray of the spinel structures and, hence, to the BeP_2_ substructure of sp-BeP_2_N_4_ (**spn** type) drawn in Fig. 18 ▸.

In summary, the phe-BeP_2_N_4_ and sp-BeP_2_N_4_ structures isolated at different pressures can be understood using the EZKC. The BeP_2_ substructure of phe-BeP_2_N_4_ [Fig. 12 ▸(*a*)**]** can be considered as an intergrowth of fragments of the cristobalite and the tridymite structures while in sp-BeP_2_N_4_ [Fig. 12 ▸(*b*)], the BeP_2_ substructure is MgCu_2_ type, characteristic of both the *AB*_2_ cubic Laves phases and the cation arrays in the spinel-type structures (Fig. 19 ▸).

### 2.10. Additional four-connected phospho­nitrides

To conclude this work, we want to mention that four-connected P skeletons also occur in many other compounds. Some of them will be described briefly next. In all of them, the P atoms are tetrahedrally coordinated by four N atoms, yielding networks with PN_2_ stoichiometry. These networks and hence the three-dimensional substructure obey the EZKC. The donor atoms are not only the cations (counterions) but also the P atoms which donate one electron each to the N atoms. The result is that the P atoms convert into Ψ-Si and the N atoms into Ψ-O, producing structures characteristic of tetrels, like in SiO_2_.

**Figure 20:**
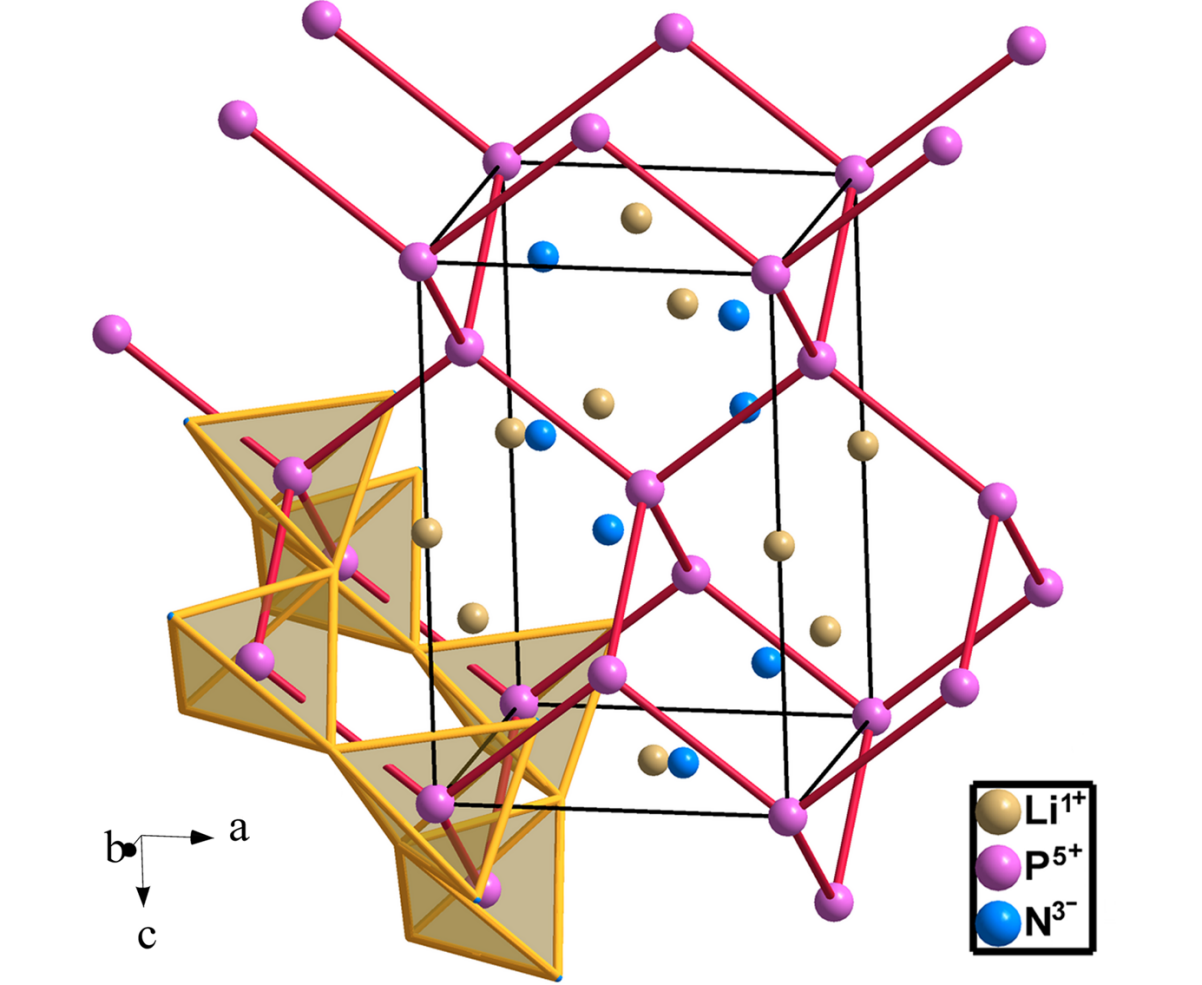
The structure of LiPN_2_. P atoms are four-connected in a diamond-like network like Si atoms in β-cristobalite. The tetrahedral coordination of the PN_4_ units is highlighted in one of the rings in chair conformation.

Compounds LiPN_2_, NaPN_2_ and CuPN_2_ (*I*42*d*, No. 122) are of the γ-LiBO_2_ type (ICSD database). Thus, the structure of LiPN_2_ (Schnick & Lücke, 1990 ▸) is an Li-filled cristobalite-like structure in which both the Li and the P atoms donate one electron each to the two N atoms, converting LiPN_2_ into Ψ-He[Ψ-SiO_2_]. The tetrahedral PN_2_ substructure has a **dia**-type P skeleton (Fig. 20 ▸).

**Figure 21:**
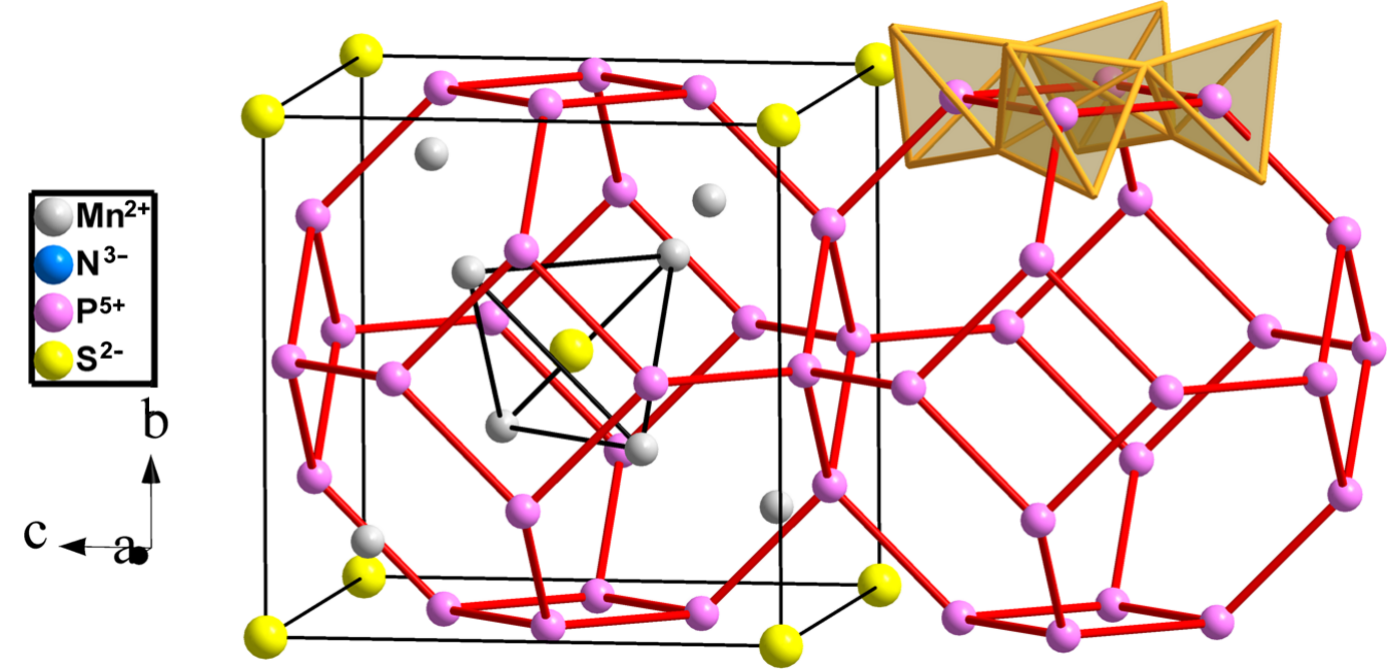
Mn-filled structure of the **sod** type formed by the P atoms in Mn_4_P_6_N_12_S, showing the four-connectivity of P atoms (Ψ-Si) and the tetrahedral coordination of Ψ-SiO_4_ units (highlighted on upper right). S atoms are located at the center of Mn_4_ tetrahedra.

Compounds Zn_7_(P_12_N_24_)Cl_2_, Zn_4_(P_2_N_4_)_3_S, Sn_6_(P_12_N_24_), Mg_4_P_6_N_12_S, Mn_4_P_6_N_12_S and Fe_4_P_6_SN_12_ are of the **sod** type (*I*43*m*, No. 217) forming a tetrahedral PN_2_ skeleton in which the P atoms are four-connected. In Mn_4_P_6_N_12_S (Griesemer *et al.*, 2021 ▸), represented in Fig. 21 ▸, the Mn and the P atoms transfer a total of (eight + six = 14 electrons). Two of them are accepted by the S atom and the remaining 12 electrons are transferred to the 12 N atoms. The formula becomes [Ψ-V][Ψ-SiO_2_][Ψ-Ar].

**Figure 22:**
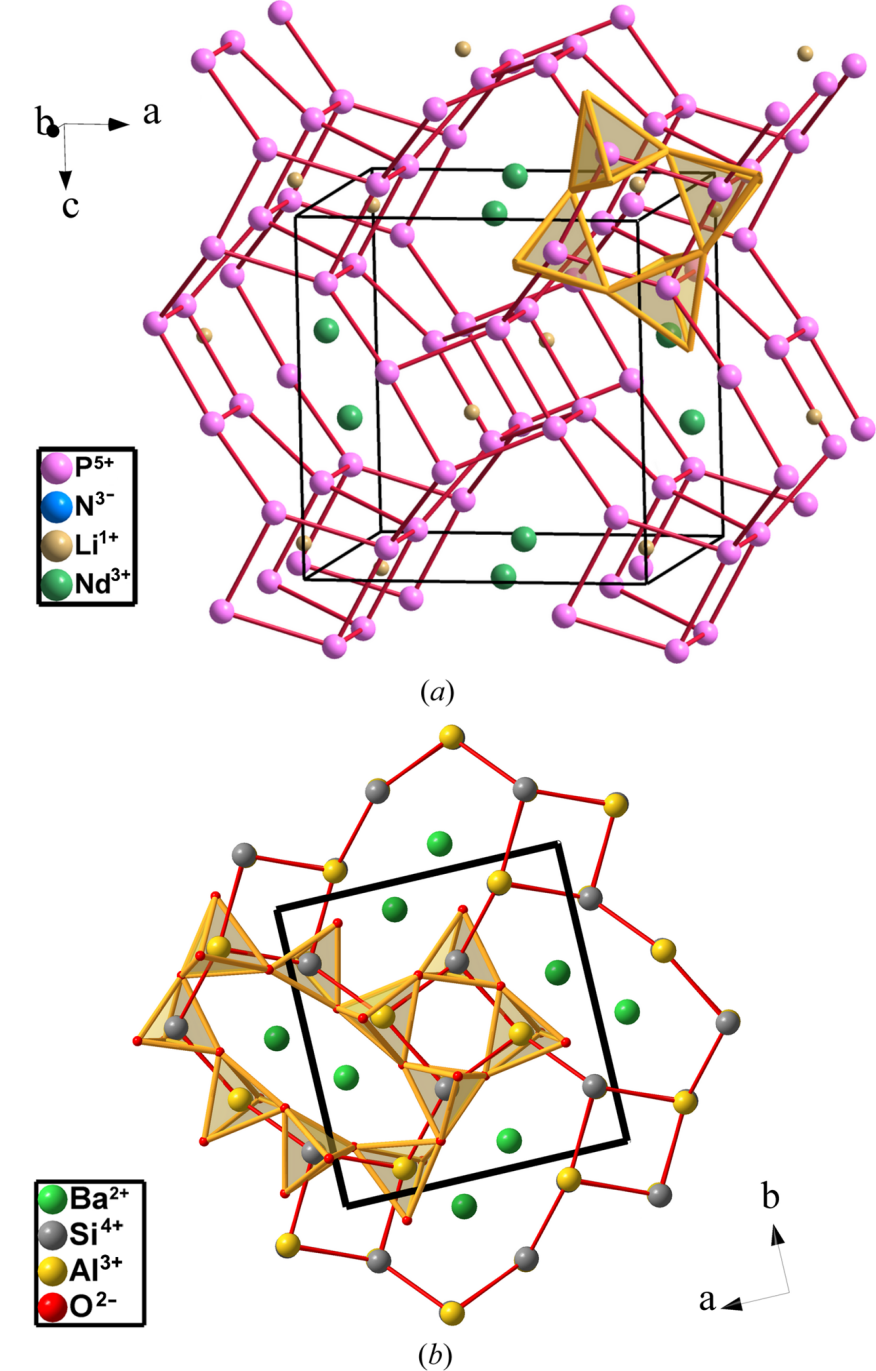
(*a*) Perspective view of the structure of LiNdP_4_N_8_ showing the four-connectivity of the P skeleton. Nd atoms occupy positions in the octagonal tunnels. Li atoms locate in the square tunnels (small ochre spheres). (*b*) Structure of paracelsian Ba[Al_2_Si_2_O_8_] projected onto the *ab* plane. The octagonal tunnels contain the Ba atoms.

LiNdP_4_N_8_ is orthorhombic (*Pnma*, No. 62) (Kloß & Schnick, 2015 ▸). The P_4_N_8_ substructure is similar to that of [Al_2_Si_2_O_8_] in paracelsian Ba[Al_2_Si_2_O_8_] as mentioned by the authors. Both structures are drawn in Fig. 22 ▸.

The P skeleton is again four-connected [see Fig. 22 ▸(*a*)] and the P atoms center the PN_4_ tetrahedra. The Nd atoms locate at the octagonal tunnels (Ba atoms in paracelsian), whereas the Li atoms occupy positions in the square tunnels. Similar tunnels are empty in paracelsian.

The four-connectivity of the P skeleton fits the EZKC. A total of eight electrons are transferred to the eight N atoms. The Li and Nd atoms donate four electrons and the four P atoms transfer four additional charges that convert LiNdP_4_N_8_ into [Ψ-He][Ψ-La][Ψ-Si_4_O_8_]. An in-depth discussion of this skeleton can be found in the book by Vegas (2018 ▸).

**Figure 23:**
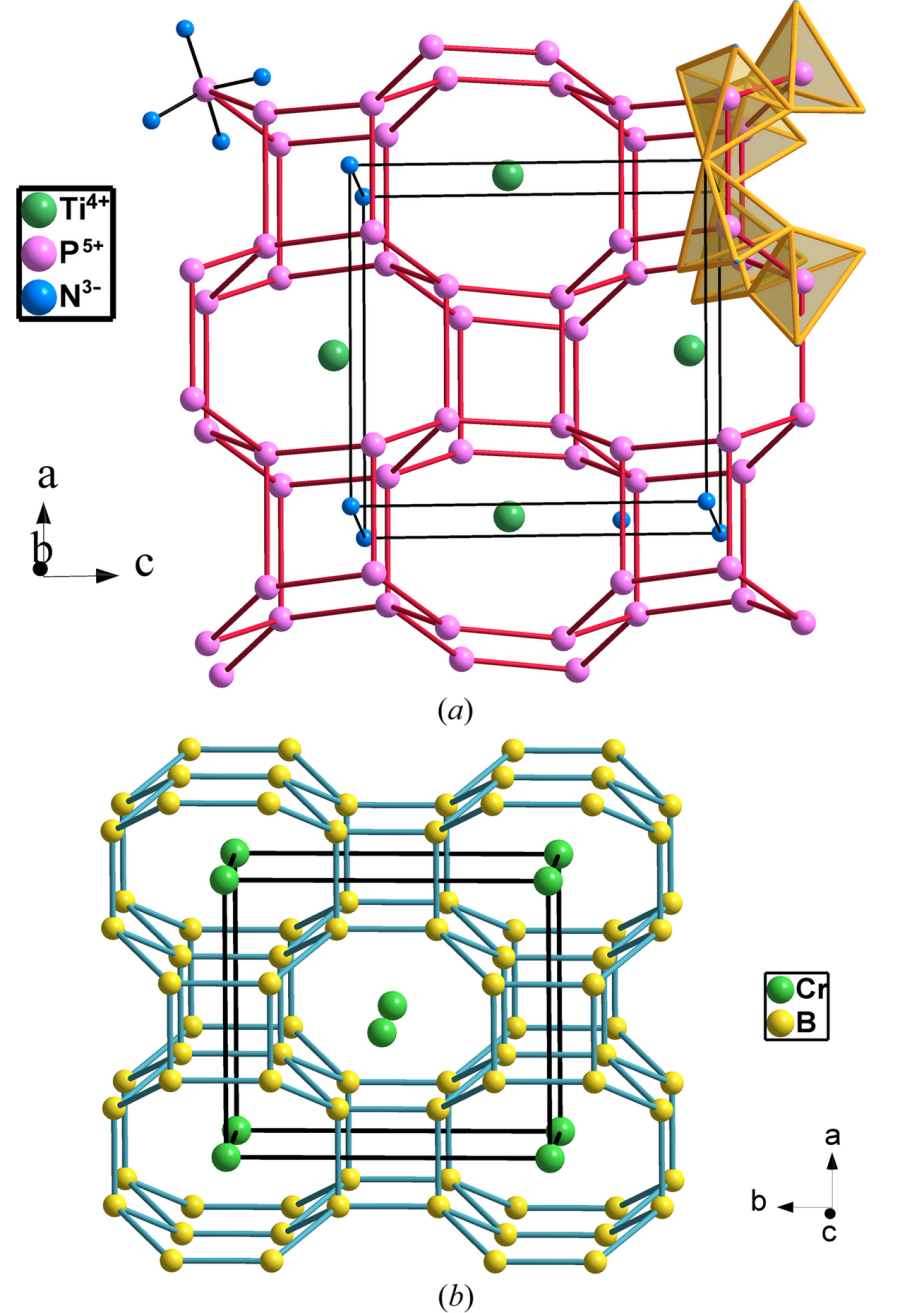
(*a*) The four-connected P skeleton in TiP_4_N_4_ in which P atoms center PN_4_ tetrahedra, highlighted in the upper part. Ti atoms are located in the octagonal tunnels. (*b*) The structure of CrB_4_ (**crb**). The charge transfer from Cr to B atoms makes B atoms adopt a Ψ-C skeleton.

TiP_4_N_8_ (*Pmn*2_1_, No. 31) was reported by Eisenburger *et al.* (2022 ▸), who described the skeleton simply as formed by four-, six- and eight-membered rings. The structure analysis with *ToposPro* (Blatov *et al.*, 2014 ▸) reveals that the [P_4_N_8_] skeleton corresponds with the zeolite BCT motif (**crb**), which is also found in CrB_4_, β-BeO as well as in the [AlP] skeleton of metavariscite AlPO_4_·2H_2_O (see Fig. 23 ▸). The four-connected skeleton of the P atoms fits the EZKC. Thus, eight electrons (four electrons from Ti and one electron from each of the four P atoms) transferred to eight N atoms convert N → Ψ-O, yielding the Ψ-formula Ψ-Ar[Ψ-Si_4_O_8_].

**Figure 24:**
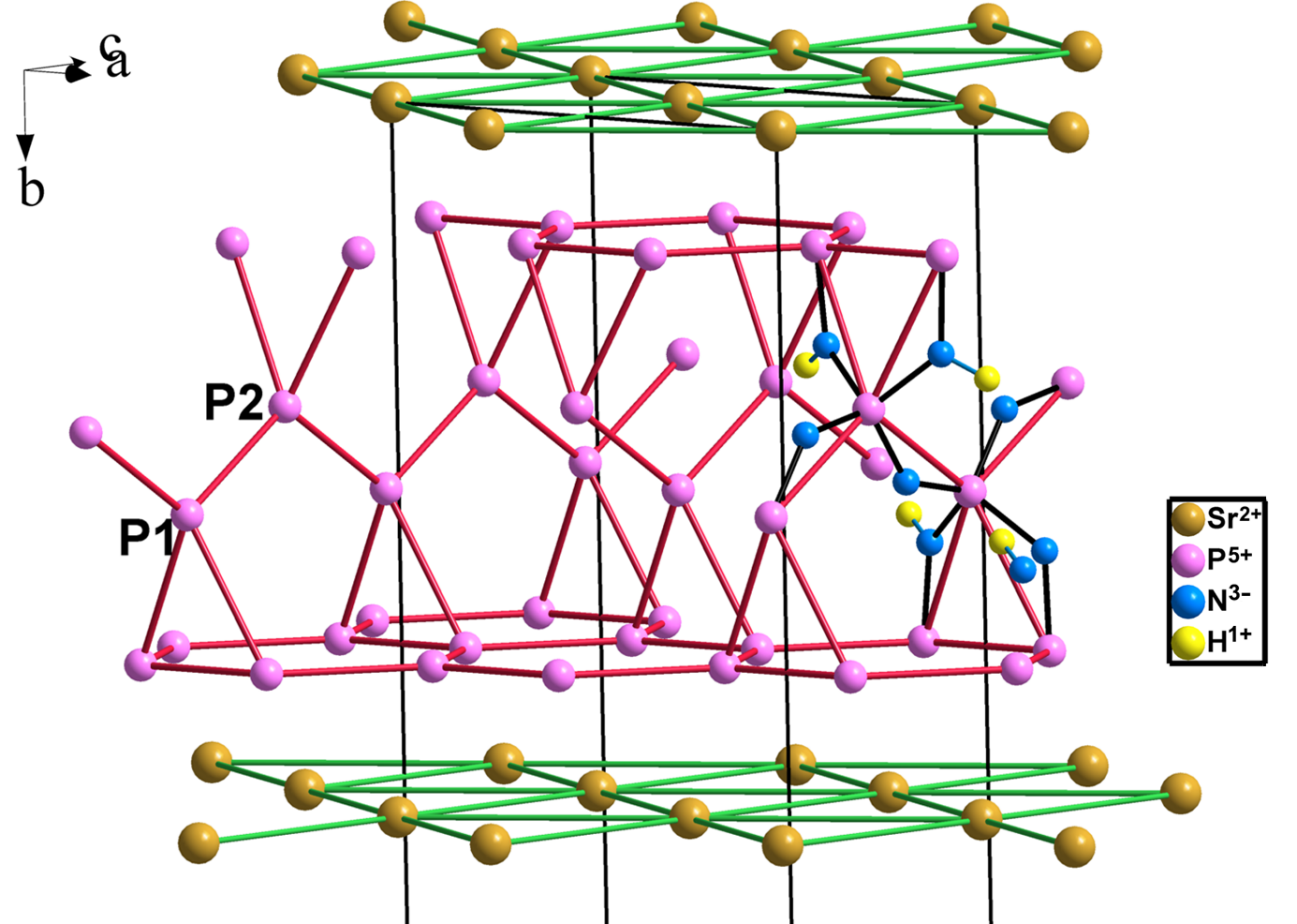
The layered structure of SrH_4_P_6_N_12_ showing the four-connected network of P atoms (violet) forming 4^2^L137 layers and their tetrahedral coordination by N(Ψ-O) atoms. Some NH groups are shown (H atoms as small yellow spheres).

In SrH_4_P_6_N_12_ (*Fmm*2, No. 42) (Wendl & Schnick, 2018 ▸), the P_6_N_12_ moiety presents a layered structure in which the PN_2_ blocks are intercalated with monoatomic 3^6^ sheets of Sr atoms. The layer net is 4^2^L137 and is represented in Fig. 24 ▸. Again, the P skeleton is four-connected with the P atoms at the center of the PN_4_ tetrahedra. The EZKC is accomplished since the Sr atoms and the six P atoms provide eight electrons which transferred to the eight N atoms converting them into Ψ-O atoms. The four H atoms are bonded to four N atoms producing NH groups (equivalent to O atoms). SrH_4_P_6_N_12_ can be reformulated as [Ψ-Kr][Ψ-SiO_2_]_6_.

## 3. Concluding remarks

In this work, we have analyzed the structures of a series of phospho­nitrides (also known as nitridophosphates) and shown that, like in the aluminates, silicates, and many other oxides, the extended Zintl–Klemm concept (EZKC) also applies to these compounds, *i.e.* their crystal structures can be rationalized and better understood in light of this concept.

This study complements a previous one (Santamaría-Pérez *et al.*, 2005 ▸) in which the EZKC was applied to the structure of the oxonitride Ce_1__6_Si_1__5_O_6_N_3__2_. In this compound, reported by Köllisch & Schnick (1999 ▸), the Si atoms present two types of coordination, *i.e.* tetrahedral Si^[4]^ and octahedral Si^[6]^, the latter being unexpected in a compound obtained at standard pressure. The application of the EZKC as well as the assumption of an amphoteric character of the Si atoms, allowed us to explain the double coordination sphere of the Si atoms. Thus, if Si^[6]^ atoms are considered as donors and tetrahedral Si^[4]^ atoms are considered as acceptors, then, the structure is fully explained. The EZKC has also been successfully applied by Vegas & Lobato (2023 ▸) to explain the structures of other closed-packed nitrides (Mg_2_PN_3_, Zn_2_PN_3_ and Ca_2_PN_3_).

The application of the EZKC to phospho­nitrides finds that the P atoms typically act as donors and N atoms as acceptors, allowing us to conclude that the name of phospho­nitrides given to these compounds is correct. A similar conclusion was reached by Contreras-García *et al.* (2020 ▸) when they studied the boron phosphate, BPO_4_, which should now be called phospho­rus borate, PBO_4_. The fact that the P atoms donate one electron to the N atoms in most structures described here, makes the P atoms convert into Ψ-Si, so explaining the tetrahedral skeletons of the P atoms within P_2_N_4_ networks. The conversion of N atoms into Ψ-O also justifies the tetrahedral coordination of P atoms (Ψ-Si), just as Si atoms do in many of the polymorphs of SiO_2_ at room pressure. As found in previous studies (Santamaría-Perez & Vegas, 2003 ▸; Santamaría-Pérez *et al.*, 2005 ▸), either O or Ψ-O atoms locate near the midpoint between Al—Al, Si—Si, or P—P bonds to capture each bonding electron pair, *i.e.* the P skeletons are converted into skeletons of the *p*-block elements obeying the 8-N rule and forming single covalent 2*c*—2*e* bonds. These explanations represent a good example of the superiority of the EZKC over the classical ionic model.

As an example, we can recall the structure of Ge^II^P_2_N_4_ whose P_2_N_4_ substructure is formed by a four-connected skeleton of P atoms, with the N atoms located close to every P⋯P contact. The four-connected skeleton, characteristic of tetrels, should not be expected for a pentel, so that the structure is better explained as due to the transfer of two electrons from Ge → two N, followed by the transfer of two more electrons from two P → two N, giving the pseudo-formula Ψ-Zn^0^Si_2_O_4_. The four connectivity of the P atoms is then justified. Moreover, the similarity of the P(Ψ-Si) skeleton (Fig. 6 ▸) with that of Al atoms (Ψ-Si) in the Zintl phase SrAl_2_ (Fig. 7 ▸) is eloquent and says that the Zintl–Klemm concept applies not only to the Zintl phases but also to both oxides and nitrides. Numerous examples have been given previously (Vegas & Jansen, 2002 ▸; Vegas, 2018 ▸) which illustrate how the nominal cations in oxides fulfill the Zintl–Klemm concept despite being embedded in an anionic matrix (either oxides or nitrides). The surprising example of the pair of compounds, BaSi and BaSiO_3_, shown in Figs. 5 ▸(*c*) and 5 ▸(*d*), is paradigmatic.

The model can be extended to other compounds containing skeletons with P_*x*_N_2*x*_ stoichiometry. We can mention the series Sr_3_P_5_N_10_Cl, Sr_3_P_5_N_10_Br, Ba_3_P_5_N_10_Cl, Ba_3_P_5_N_10_Br and Ba_3_P_5_N_10_I, with orthorhombic symmetry (*Pnma*, No. 62). Analysis using the *ToposPro* package (Blatov *et al.*, 2014 ▸) indicates that they are isostructural to the zeolite JOZ whose PN_2_ tetrahedral substructure contains four-connected P atoms (Ψ-Si), according to the EZKC. Similarly, in SrP_3_N_5_(NH) (*P*2_1_/*c*, No. 14), the P atoms are four-connected and the P_3_N_5_(NH) substructure, equivalent to P_3_N_5_O, is similar to other silica structures of the topological type 4^3^T281. After the EZKC, the transfer of two electrons from Sr → two N atoms and three electrons from three P → three N, gives the pseudo-formula Ψ-Kr[Ψ-SiO_2_]. The reader may visualize the skeletons of these compounds for further examples of how the EZKC can account for the connectivity of the P skeletons, an aspect of the crystal structures that is not considered when the structures are regarded as the result of interactions between nominal anions and cations as done by the traditional ionic model.

## Figures and Tables

**Table 1:** Phosphonitrides fitting the extended Zintl–Klemm concept

Phospho­nitride section	Pseudo-formula	Topology of anion network†	ICSD reference code	Reference
(AE)_2_AlP_8_N_15_(NH) (AE = Ca, Sr, Ba) (Section 2.1)	(Ψ-Kr)_2_(Ψ-Ne)[Ψ-Si_8_O_16_]	4^4^T170	–	Pointner *et al.* (2024 ▸)
Ge^IV^PN_3_ (Section 2.2)	(Ψ-Ar)[Ψ-SO_3_]	2C1	176671	Ambach *et al.* (2024 ▸)
Ge^II^P_2_N_4_ (Section 2.3)	(Ψ-Zn)[Ψ-Si_2_O_4_]	**sra**	176669	Ambach *et al.* (2024 ▸)
CaP_2_N_4_, SrP_2_N_4_ (Section 2.4)	(Ψ-Kr)[Ψ-Si_2_O_4_]	**tpd**	416930	Karau *et al.* (2007 ▸)
BaP_2_N_4_ (Section 2.5)	(Ψ-Xe)[Ψ-Si_2_O_4_]	**cbo**	651183	Karau & Schnick (2005 ▸)
phe-BeP_2_N_4_ (*R*3) (Sections 2.6, 2.7)	(Ψ-He)[Ψ-Si_2_O_4_]	**lcs**	40797	Vogel *et al.* (2020 ▸)
sp-BeP_2_N_4_ (*Fd*3*m*) (Sections 2.7, 2.9)	(Ψ-He)[Ψ-Si_2_O_4_]	**spn**	40805	Vogel *et al.* (2020 ▸)
LiGaGe (Section 2.8)	(Ψ-He)[Ψ-Si]Si	**lon**	25310	Bockelmann & Schuster (1974 ▸)
LiGaGeO_4_ (Section 2.8)	(Ψ-He)[Ψ-Si_2_O_4_]	**lcs**	67239	Hartman (1989 ▸)
LiPN_2_ (Section 2.10.1)	(Ψ-He)[Ψ-SiO_2_]	**dia**	66007	Schnick & Lücke (1990 ▸)
Mn_4_P_6_N_12_S (Section 2.10.2)	[Ψ-V][Ψ-SiO_2_]_6_[Ψ-Ar]	**sod**	138799	Griesemer *et al.* (2021 ▸)
LiNdP_4_N_8_ (Section 2.10.3)	[Ψ-He][Ψ-La][Ψ-SiO_2_]_4_	**pcl**	429542	Kloß & Schnick (2015 ▸)
TiP_4_N_8_ (Section 2.10.4)	[Ψ-Ar][Ψ-SiO_2_]_4_	**crb** BCT	46202	Eisenburger *et al.* (2022 ▸)
SrH_4_P_6_N_12_ (Section 2.10.5)	[Ψ-Kr][H^+^_4_][Ψ-SiO_2_]_6_	4^2^L137	434261	Wendl & Schnick (2018 ▸)
SrP_3_N_5_(NH) (Section 3)	[Ψ-Kr][Ψ-SiO_2_]_3_	4^3^T281	434532	Vogel & Schnick (2018 ▸)
Sr_3_P_5_N_10_Cl	[Ψ-Kr][Ψ-SiO_2_]_5_[Ψ-Ar]	JOZ	143745	Wendl *et al.* (2021 ▸)
Sr_3_P_5_N_10_Br			143746	Wendl *et al.* (2021 ▸)
Ba_3_P_5_N_10_Cl			238968	Marchuk *et al.* (2015 ▸)
Ba_3_P_5_N_10_Br			428381	Marchuk & Schnick (2015 ▸)
Ba_3_P_5_N_10_I (Section 3)			238969	Marchuk *et al.* (2015 ▸)

† Topology names are given according to the Reticular Chemistry Structure Resource (RCSR) nomenclature (lowercase bold three-letter symbols) (O’Keeffe *et al.*, 2008 ▸), International Zeolite Association (IZA) nomenclature (uppercase three-letter symbols) and *ToposProN*D*n* nomenclature (Blatov *et al.*, 2021 ▸). In the *N*D*n* nomenclature, *N* designates the sequence of CNs of all inequivalent nodes of the net, D denotes the net periodicity (C, L or T for one-, two- or three-periodic nets) and *n* enumerates different topologies with the same *N*D symbol.

## References

[bb1] Alcock, N. W., Evans, D. A. & Jenkins, H. D. B. (1973). *Acta Cryst.* B**29**, 360–361.

[bb2] Ambach, S. J., Krach, G., Bykova, E., Witthaut, K., Giordano, N., Bykov, M. & Schnick, W. (2024). *Inorg. Chem.* **63**, 8502–8509. 10.1021/acs.inorgchem.4c01202 38657029

[bb3] Ambach, S. J., Somers, C., de Boer, T., Eisenburger, L., Moewes, A. & Schnick, W. (2023). *Angew. Chem. Int. Ed.* **62**, e202215393. 10.1002/anie.202215393 PMC10107938 36350660

[bb64] Bader, R. F. W. (1985). *Acc. Chem. Res.* **18**, 9–15.

[bb4] Blatov, V. A., Alexandrov, E. V. & Shevchenko, A. P. (2021). *Compr. Coord. Chem. III,* Vols. **1–9**, 389–412. Elsevier.

[bb5] Blatov, V. A., Shevchenko, A. P. & Proserpio, D. M. (2014). *Cryst. Growth Des.* **14**, 3576–3586.

[bb6] Bockelmann, W. & Schuster, H. (1974). *Z. Anorg. Allg. Chem.* **410**, 233–240.

[bb7] Boudemagh, D., Fruchart, D., Haettel, R., Hlil, E. K., Lacoste, A., Ortega, L., Skryabina, N., Toboła, J. & Wolfers, P. (2011). *Solid State Phenom.* **170**, 253–258.

[bb8] Cascales, C., Gutiérrez-Puebla, E., Monge, M. A. & Ruíz-Valero, C. (1998). *Angew. Chem. Int. Ed.* **37**, 129–131. 10.1002/(sici)1521-3773(19990816)38:16<2436::aid-anie2436>3.0.co;2-w 10458814

[bb10] Contreras-García, J., Izquierdo-Ruiz, F., Marqués, M. & Manjón, F. J. (2020). *Acta Cryst.* A**76**, 197–205. 10.1107/S2053273319016826 32124857

[bb11] de Boer, T., Somers, C., Boyko, T., Ambach, S., Eisenburger, L., Schnick, W. & Moewes, A. (2023). *J. Mater. Chem. A* **11**, 6198–6204. 10.1002/anie.202215393 PMC10107938 36350660

[bb12] Eisenburger, L., Weippert, V., Paulmann, C., Johrendt, D., Oeckler, O. & Schnick, W. (2022). *Angew. Chem. Int. Ed.* **61**, 1–5, e202202014. 10.1002/anie.202202014 PMC9310718 35179291

[bb13] Griesemer, S. D., Ward, L. & Wolverton, C. (2021). *Phys. Rev. Mater.* **5**, 105003.

[bb15] Hartman, P. (1989). *Z. Kristallogr.* **187**, 139–143.

[bb16] Karau, F. W. & Schnick, W. (2005). *J. Solid State Chem.* **178**, 135–141.

[bb17] Karau, F. W., Seyfarth, L., Oeckler, O., Senker, J., Landskron, K. & Schnick, W. (2007). *Chem. Eur. J.* **13**, 6841–6852. 10.1002/chem.200700216 17566130

[bb62] Kaupp, M. (2014). In *The Chemical Bond: Fundamental Aspects of Chemical Bonding*, edited by G. Frenking & S. Shaik, ch. 1. Weinheim: Wiley-VCH.

[bb18] Kloß, S. D. & Schnick, W. (2015). *Angew. Chem. Int. Ed.* **54**, 11250–11253. 10.1002/anie.201504844 26352033

[bb19] Köllisch, K. & Schnick, W. (1999). *Angew. Chem. Int. Ed.* **38**, 357–359. 10.1002/(SICI)1521-3773(19990201)38:3<357::AID-ANIE357>3.0.CO;2-D 29711659

[bb20] Léger, J. M., Haines, J., de Oliveira, L. S., Chateau, C., Le Sauze, A., Marchand, R. & Hull, S. (1999). *J. Phys. Chem. Solids* **60**, 145–152.

[bb21] Liebau, F. (1999). *Angew. Chem. Int. Ed.* **38**, 1733–1737. 10.1002/(SICI)1521-3773(19990614)38:12<1733::AID-ANIE1733>3.0.CO;2-5 29711195

[bb22] Marchuk, A. & Schnick, W. (2015). *Angew. Chem. Int. Ed.* **54**, 2383–2387. 10.1002/anie.201410528 25573329

[bb23] Marchuk, A., Wendl, S., Imamovic, N., Tambornino, F., Wiechert, D., Schmidt, P. J. & Schnick, W. (2015). *Chem. Mater.* **27**, 6432–6441.

[bb24] Marezio, M., Remeika, J. P. & Dernier, P. D. (1969). *Acta Cryst.* B**25**, 965–970.

[bb25] Nelson, R., Ertural, C., George, J., Deringer, V. L., Hautier, G. & Dronskowski, R. (2020). *J. Comput. Chem.* **41**, 1931–1940. 10.1002/jcc.26353 32531113

[bb26] O’Keeffe, M. & Hyde, B. G. (1981). *The Role of Nonbonded Forces in Crystals*. In *Structure and Bonding in Crystals* edited by M. O’Keeffe and A. Navrotsky, Vol. I, ch. 10. New York: Wiley.

[bb61] O’Keeffe, M. & Hyde, B. G. (1985). *Struct. Bond*, **61**, 77–144.

[bb27] O’Keeffe, M., Peskov, M. A., Ramsden, S. J. & Yaghi, O. M. (2008). *Acc. Chem. Res.* **41**, 1782–1789. 10.1021/ar800124u 18834152

[bb28] Otero-de-la-Roza, A., Blanco, M. A., Pendás, A. M. & Luaña, V. (2009). *Comput. Phys. Commun.* **180**, 157–166.

[bb29] Otero-de-la-Roza, A., Johnson, E. R. & Luaña, V. (2014). *Comput. Phys. Commun.* **185**, 1007–1018.

[bb30] Parfitt, D. C., Keen, D. A., Hull, S., Crichton, W. A., Mezouar, M., Wilson, M. & Madden, P. A. (2005). *Phys. Rev. B* **72**, 054121.

[bb31] Pointner, M. M., Pritzl, R. M., Albrecht, J. M., Blahusch, L., Wright, J. P., Bright, E. L., Giacobbe, C., Oeckler, O. & Schnick, W. (2024). *Chem. A Eur. J.* **30**, e202400766. 10.1002/chem.202400766 38483015

[bb32] Pucher, F. J., Marchuk, A., Schmidt, P. J., Wiechert, D. & Schnick, W. (2015). *Chem. A Eur. J.* **21**, 6443–6448. 10.1002/chem.201500047 25765825

[bb33] Pucher, F. J., Römer, S. R., Karau, F. W. & Schnick, W. (2010). *Chem. Eur. J.* **16**, 7208–7214. 10.1002/chem.201000153 20461832

[bb34] Rieger, W. & Parthé, E. (1967). *Acta Cryst.* **22**, 919–922.

[bb35] Santamaría-Pérez, D. & Vegas, A. (2003). *Acta Cryst.* B**59**, 305–323. 10.1107/s0108768103005615 12761402

[bb36] Santamaría-Pérez, D., Vegas, A. & Liebau, F. (2005). *Struct. Bond.* **118**, 121–177. Berlin, Heidelberg: Springer.

[bb37] Sasaki, S., Prewitt, C. T., Sato, Y. & Ito, E. (1982). *J. Geophys. Res.* **87**, 7829–7832.

[bb39] Schnick, W. & Lücke, J. (1990). *Z. Anorg. Allg. Chem.* **588**, 19–25.

[bb40] Shevchenko, A. P., Shabalin, A. A., Karpukhin, I. Y. & Blatov, V. A. (2022). *Sci. Technol. Adv. Mater. Methods* **2**, 250–265.

[bb41] Vegas, A. (2011). *Struct. Bond.* **138**, 133–198.

[bb42] Vegas, A. (2018). *Structural Models of Inorganic Crystals: From the Elements to the Compounds.* Editorial de la Universitat Politècnica de València: Valencia. ISBN 978-84-90486-02-3. Spanish version: *Modelos Estructurales de Cristales Inorgánicos: De los Elementos a los compuestos*. ISBN 978-84-18465-02-4 (2019). The Spanish e-book version is available for free from the Repository of the Universidad de Burgos: Burgos (Spain) via https://hdl.handle.net/10259/5647.

[bb43] Vegas, A. & García-Baonza, V. (2007). *Acta Cryst.* B**63**, 339–345. 10.1107/S0108768107019167 17507745

[bb44] Vegas, A. & Jansen, M. (2002). *Acta Cryst.* B**58**, 38–51. 10.1107/s0108768101019310 11818653

[bb45] Vegas, A. & Jenkins, H. D. B. (2017). *Acta Cryst.* B**73**, 94–100.

[bb46] Vegas, A. & Lobato, A. (2023). In *Comprehensive Inorganic Chemistry III*, 3rd ed., pp. 51–73. ScienceDirect.

[bb60] Vegas, A., Martin, R. L. & Bevan, D. J. M. (2009). *Acta Cryst.* B**65**, 11–21. 10.1107/S010876810803423X PMC2628973 19155554

[bb47] Vogel, S., Bykov, M., Bykova, E., Wendl, S., Kloß, S. D., Pakhomova, A., Chariton, S., Koemets, E., Dubrovinskaia, N., Dubrovinsky, L. & Schnick, W. (2019). *Angew. Chem. Int. Ed.* **58**, 9060–9063. 10.1002/anie.201902845 31020764

[bb48] Vogel, S., Bykov, M., Bykova, E., Wendl, S., Kloß, S. D., Pakhomova, A., Dubrovinskaia, N., Dubrovinsky, L. & Schnick, W. (2020). *Angew. Chem. Int. Ed.* **59**, 2730–2734. 10.1002/anie.201910998 PMC7027884 31596046

[bb49] Vogel, S. & Schnick, W. (2018). *Chem. A Eur. J.* **24**, 14275–14281. 10.1002/chem.201803210 30004596

[bb50] Wendl, S. & Schnick, W. (2018). *Chem. A Eur. J.* **24**, 15889–15896. 10.1002/chem.201803125 30136742

[bb51] Wendl, S., Zipkat, M., Strobel, P., Schmidt, P. J. & Schnick, W. (2021). *Angew. Chem. Int. Ed.* **60**, 4470–4473. 10.1002/anie.202012722 PMC7985876 33201554

